# Microglia-specific deletion of histone deacetylase 3 promotes inflammation resolution, white matter integrity, and functional recovery in a mouse model of traumatic brain injury

**DOI:** 10.1186/s12974-022-02563-2

**Published:** 2022-08-06

**Authors:** Yongfang Zhao, Hongfeng Mu, Yichen Huang, Sicheng Li, Yangfan Wang, R. Anne Stetler, Michael V. L. Bennett, C. Edward Dixon, Jun Chen, Yejie Shi

**Affiliations:** 1 Pittsburgh Institute of Brain Disorders & Recovery and Department of Neurology, University of Pittsburgh, Pittsburgh, PA 15213 USA; 2 Dominick P. Purpura Department of Neuroscience, Albert Einstein College of Medicine, Bronx, NY 10461 USA; 3 Department of Neurosurgery, University of Pittsburgh, Pittsburgh, PA 15213 USA; 4 Geriatric Research, Education and Clinical Center, Veterans Affairs Pittsburgh Health Care System, Pittsburgh, PA 15261 USA

**Keywords:** Conditional gene knockout, Controlled cortical impact, HDAC3, Neuroinflammation

## Abstract

**Background:**

Histone deacetylases (HDACs) are believed to exacerbate traumatic brain injury (TBI) based on studies using pan-HDAC inhibitors. However, the HDAC isoform responsible for the detrimental effects and the cell types involved remain unknown, which may hinder the development of specific targeting strategies that boost therapeutic efficacy while minimizing side effects. Microglia are important mediators of post-TBI neuroinflammation and critically impact TBI outcome. HDAC3 was reported to be essential to the inflammatory program of in vitro cultured macrophages, but its role in microglia and in the post-TBI brain has not been investigated in vivo.

**Methods:**

We generated HDAC3^LoxP^ mice and crossed them with CX3CR1^CreER^ mice, enabling in vivo conditional deletion of HDAC3. Microglia-specific HDAC3 knockout (HDAC3 miKO) was induced in CX3CR1^CreER^:HDAC3^LoxP^ mice with 5 days of tamoxifen treatment followed by a 30-day development interval. The effects of HDAC3 miKO on microglial phenotype and neuroinflammation were examined 3–5 days after TBI induced by controlled cortical impact. Neurological deficits and the integrity of white matter were assessed for 6 weeks after TBI by neurobehavioral tests, immunohistochemistry, electron microscopy, and electrophysiology.

**Results:**

HDAC3 miKO mice harbored specific deletion of HDAC3 in microglia but not in peripheral monocytes. HDAC3 miKO reduced the number of microglia by 26%, but did not alter the inflammation level in the homeostatic brain. After TBI, proinflammatory microglial responses and brain inflammation were markedly alleviated by HDAC3 miKO, whereas the infiltration of blood immune cells was unchanged, suggesting a primary effect of HDAC3 miKO on modulating microglial phenotype. Importantly, HDAC3 miKO was sufficient to facilitate functional recovery for 6 weeks after TBI. TBI-induced injury to axons and myelin was ameliorated, and signal conduction by white matter fiber tracts was significantly enhanced in HDAC3 miKO mice.

**Conclusion:**

Using a novel microglia-specific conditional knockout mouse model, we delineated for the first time the role of microglial HDAC3 after TBI in vivo. HDAC3 miKO not only reduced proinflammatory microglial responses, but also elicited long-lasting improvement of white matter integrity and functional recovery after TBI. Microglial HDAC3 is therefore a promising therapeutic target to improve long-term outcomes after TBI.

**Supplementary Information:**

The online version contains supplementary material available at 10.1186/s12974-022-02563-2.

## Background

Severe traumatic brain injury (TBI) is a devastating condition that imposes a huge economic burden on society, yet there remains no effective therapy that can stop the progressive brain damage and neurological deficits after TBI. Recent years have witnessed significant advances in preclinical TBI research that has identified numerous therapeutic agents showing promising efficacy in treating TBI, one cluster of them being the inhibitors of histone deacetylases (HDACs). HDACs are a superfamily of enzymes that can reduce acetylation of histones and exert transcriptional repression [1]. The balance between the antagonistic actions of HDACs and histone acetyltransferases determines histone acetylation and gene expression, thereby regulating a variety of physiological and pathological cellular processes [1]. Notably, previous studies using HDAC inhibitors to treat TBI have consistently observed improvement in TBI outcomes [2–5]. ITF2357, a pan-HDAC inhibitor, reduced neuronal death and lesion volume in a mouse model of closed head injury [2]. More recently, another two inhibitors of Class I and II HDACs, valproic acid and scriptaid, were also found to exert anti-apoptotic and anti-inflammatory effects after TBI and improve long-term neurological functions [3–5]. Despite these promising therapeutic effects of HDAC inhibitors, it remains unknown which HDAC isoform is the major player exacerbating brain injury after TBI, owing to the pan-targeting nature of these inhibitors. Furthermore, the primary cellular mechanism responsible for improved outcome upon HDAC inhibition cannot be inferred, as systemically administered HDAC inhibitors would act on many types of cells. These limitations of existing studies hinder the development of more specific targeting strategies that may boost efficacy and reduce side effects compared to using pan-HDAC inhibitors.

TBI evokes a plethora of complex injury responses consisting of immediate and irreversible mechanical damage and delayed secondary injuries. A key mechanism of secondary brain injury after TBI is neuroinflammation, which evolves during the subacute phase and critically impacts long-term TBI outcome [6–8]. Being the brain’s resident immune cells, microglia are major regulators of post-TBI neuroinflammation through their highly plastic functions [9–11]. Proinflammatory microglial responses are typically linked to deterioration after TBI, whereas inflammation-resolving microglial responses are believed to facilitate post-TBI brain repair and functional recovery [4, 12, 13]. One unanswered question, however, is the relative contribution of microglia versus monocyte-derived macrophages—the blood counterpart of microglia—that enter the brain after TBI, because commonly used markers do not distinguish activated microglia from macrophages. On the other hand, whether weaker proinflammatory microglial responses are the driving force or simply a result of smaller TBI lesions is also unknown, due to the lack of cell-specific targeting approaches. Interestingly, HDAC3 was recently identified to be a potent regulator of immune cell phenotype, whereby in vitro cultured HDAC3-null bone marrow-derived macrophages failed to activate nearly half of the inflammatory gene expression program when stimulated by lipopolysaccharides [14]. Depleting HDAC3 could therefore foster beneficial responses of immune cells, such as microglia, and thereby improve the outcome after TBI.

To date, little is known about the role of HDAC3 in the pathophysiology of TBI, attributed in part to the lack of tools to *specifically* manipulate this HDAC in vivo. Deletion of HDAC3 in the germline leads to early embryonic lethality [15], and no study has tested the effect of *selective* HDAC3 inhibitors against TBI so far. To fill this knowledge gap, we engineered HDAC3^LoxP^ mice which enable conditional deletion of HDAC3 in adults and in selected cell types, thus avoiding developmental impact caused by early universal HDAC3 deficiency. The present study aimed to investigate the role of HDAC3 in microglial responses after TBI, and utilized this novel conditional HDAC3 knockout mouse model to specifically ablate HDAC3 in microglia.

## Methods

Key resources that are essential to reproduce the results are provided in Additional file 1: Table S1.

### Animals

**Fig. 1:**
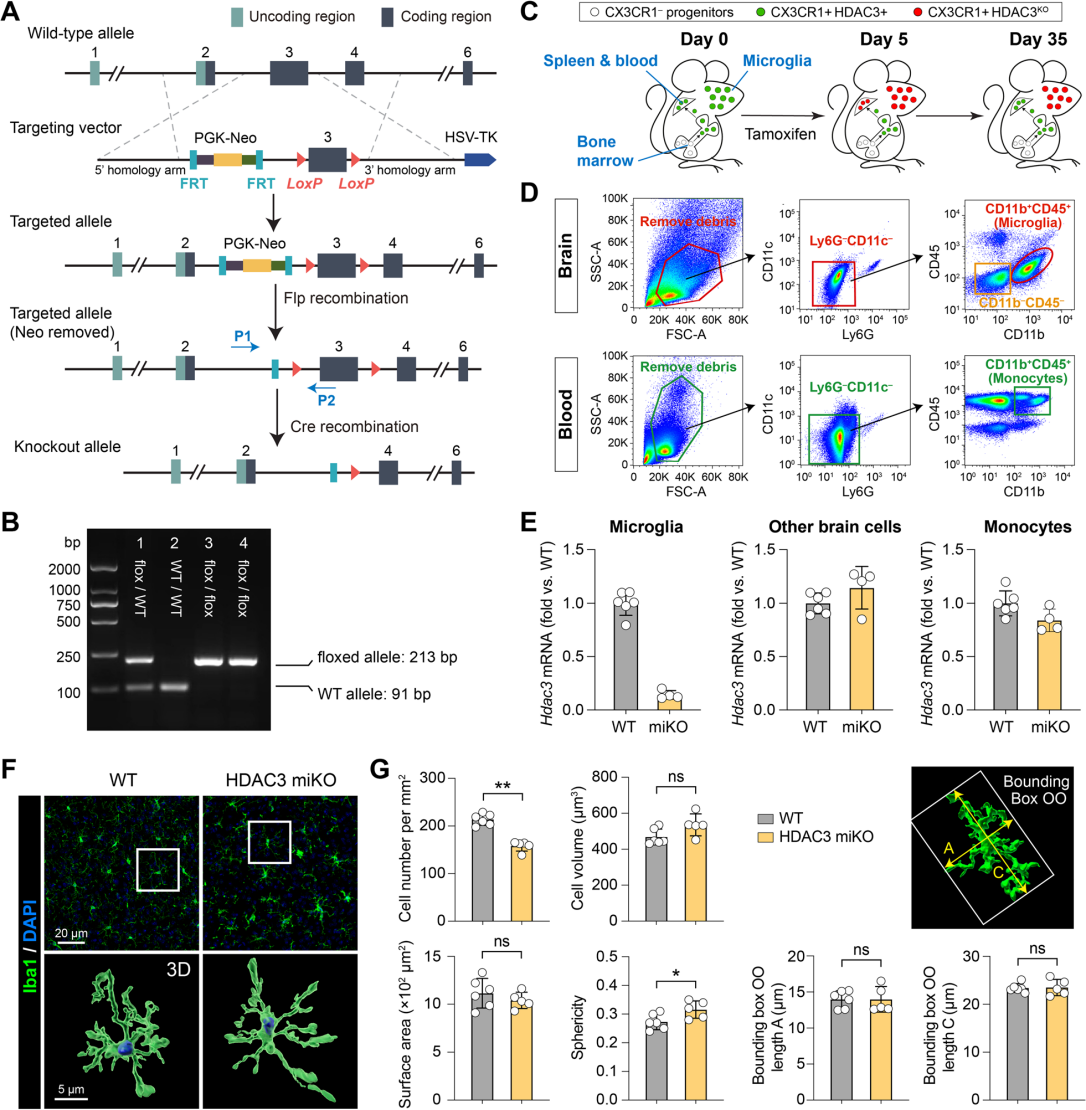
Generation and characterization of microglia-specific HDAC3 knockout mice. **A** Targeting strategy to generate HDAC3^LoxP^ mice with *LoxP*-flanked exon 3 of the *Hdac3* gene. In the presence of Cre, the *LoxP* sites recombine and delete the floxed region of *Hdac3*. P1 and P2, genotyping primers. **B** PCR genotyping demonstrating generation of heterozygous mice (lane 1) and subsequent breeding to obtain homozygous mice containing the *LoxP* sites on both alleles (lanes 3 and 4). Using primers flanking the 5′ *LoxP* site, the floxed allele runs at a weight of 213 bp, whereas the wild-type (WT) allele has a weight of 91 bp. **C** Pulse knockout strategy to restrict tamoxifen-induced HDAC3 KO to microglia. Peripheral CX3CR1^+^ cells are continuously replaced from CX3CR1^–^ progenitor cells in the bone marrow. HDAC3 is deleted 5 days after tamoxifen treatment in both microglia and peripheral CX3CR1^+^ cells. After another 30 days, peripheral CX3CR1^+^ cells are replenished from bone marrow progenitors by new CX3CR1^+^ cells that have not undergone recombination. Therefore, only microglia are HDAC3 KO, and most peripheral monocytes express HDAC3. **D** FACS gating strategy for brain microglia (CD11b^+^CD45^+^), other brain cells (CD11b^–^CD45^–^), and blood monocytes (CD11b^+^CD45^+^). **E** Quantitative PCR was performed 30 days after tamoxifen treatment on FACS-sorted cells to verify the deletion of HDAC3 in microglia but not in other brain cells or blood monocytes. *n* = 6 (WT) and 4 (HDAC3 miKO) mice. **F**, **G** The number and morphology of microglia were assessed in the brain of HDAC3 miKO mice and WT control mice after Iba1 immunostaining and 3D rendering of images. *n* = 6 mice (237 cells) for WT. *n* = 5 mice (140 cells) for HDAC3 miKO. Shown are the mean ± SD. *p < 0.05, **p < 0.01. *ns* no significant difference

We generated HDAC3^LoxP^ mice harboring a conditional null allele of *Hdac3* using previously described methodology [16]. Targeting of *Hdac3* was achieved by introducing *LoxP* sites upstream and downstream of exon 3 through homologous recombination (Fig. 1A). The targeting vector consisted of a 4.1-kb 5′ homology arm, an FRT-flanked PGK-Neo cassette, *LoxP*-flanked exon 3, a 3.9-kb 3′ homology arm, and an HSV-TK gene at the 3′ end of the recombination regions for negative selection (Fig. 1A). The construct was linearized and electroporated into mouse embryonic stem (ES) cells, and the resulting cells were subjected to positive and negative drug selection by neomycin (G418) and ganciclovir, respectively. Drug-selected, homologously recombined ES cells were confirmed by Southern analysis. The targeted ES cell clones were injected into C57BL/6 blastocysts, and two male chimeras with germline transmission were obtained. The chimeras were then bred with C57BL/6J female mice to obtain ES cell-derived offspring (F1). The resulting F1 mice were verified heterozygous for the floxed *Hdac3* gene by sequencing the PCR products. The mice were then bred with Flp-expressing mice to remove the Neo cassette, followed by further breeding with C57BL/6J mice to remove the Flp gene. The offspring were assessed by PCR to confirm the presence of the floxed alleles (Fig. 1B and Additional file 2).

Microglia-specific HDAC3 knockout (HDAC3 miKO) mice were obtained by crossing the CX3CR1^CreER^ mice [17] and HDAC3^LoxP^ mice for two generations. To induce gene deletion, HDAC3 miKO mice (genotype: *Cx3cr1*^*CreER/wt*^*:Hdac3*^*flox/flox*^) received intraperitoneal injections of tamoxifen (75 mg/kg daily for 5 days). Homozygous HDAC3^LoxP^ mice served as age- and sex-matched wild-type (WT) control mice for the HDAC3 miKO mice, and received the same tamoxifen treatments. Mice were subjected to TBI 30 days after tamoxifen treatments. Both the homozygous HDAC3^LoxP^ mice and HDAC3 miKO mice were viable, fertile, and did not exhibit any gross physical or behavioral abnormalities. All mice were housed in a specific pathogen-free facility with a 12-h light/dark cycle. Food and water were available ad libitum. Adult male mice at 12–16 weeks of age (20–30 g body weight) were used in this study. Experimental group assignments were randomized with a lottery-drawing box, and surgeries and all outcome assessments were performed by investigators blinded to mouse genotype and experimental group assignment whenever possible. A total of 94 WT mice and 86 HDAC3 miKO mice were used in this study. Only those animals that died during or shortly after the controlled cortical impact surgery (1 WT and 3 HDAC3 miKO mice) were excluded from further analyses. There was no outlier excluded from data analyses. Detailed information on the animals used in this study and timepoints of all outcome measurements are provided in Additional file 1: Table S2. The type and amount of tissue samples harvested for each outcome parameter are summarized in Additional file 1: Table S3.

### Traumatic brain injury model

TBI was induced in mice by controlled cortical impact (CCI) to the right brain hemisphere as previously described [18]. Briefly, anesthesia was induced in mice with 5% isoflurane in a 67% N_2_O/30% O_2_ mixture, and was maintained with 1.5% isoflurane during surgery. The head of the mouse was stabilized in a stereotaxic frame and a skin incision was made to expose the skull. A right parietal craniotomy (centered 0.5 mm anterior and 2.0 mm lateral to bregma; diameter: 3.5 mm) was prepared to expose the dura and cerebral cortex. CCI was performed with a pneumatically driven CCI device (Precision Systems and Instrumentation), which used a flat-tipped impactor (diameter: 3 mm) to compress the exposed brain tissue to a depth of 1.5 mm (dwell time: 150 ms; peak velocity: 3.5 m/s). Rectal temperature was maintained at 37 ± 0.5 °C using a heating pad during surgery. Animals that were subjected to anesthesia and skin incision but did not receive craniotomy preparation or CCI served as non-injury baseline controls.

### Neurobehavioral tests

#### Body curl test

The body curl test was performed as previously described [19] to assess contralateral torso flexion. Briefly, mice were hand-suspended by the tail and scored for the degree of torso flexion from vertical towards the contralesional side according to the following criteria: (1) deviating 0° from vertical (no flexion); (2) flexion of the torso 22.5° or less from vertical; (3) flexion between 22.5° and 45° from vertical; (4) flexion 45° or more and with or without grasping of the hindlimbs by forelimbs. Three trials were performed on each testing day and the scores were averaged.

#### Hanging wire test

The hanging wire test was performed as described previously [4] to assess the condition and strength of muscles. Mice were placed on the midway of a stainless-steel bar (length: 50 cm; diameter: 2 mm) resting on 2 vertical supports and elevated 37 cm above a flat surface, and were observed for 30 s. The animal’s performance was scored as follows: 0, fell off; 1, hung onto the bar with two forepaws; 2, hung onto the bar with added attempt to climb onto the bar; 3, hung onto the bar with two forepaws and one or both hind paws; 4, hung onto the bar with all four paws and with tail wrapped around the bar; 5, escaped to one of the supports. Animals were trained for 3 days before the CCI surgery. After surgery, this test was performed for 35 days. Three trials were performed on each testing day.

#### Adhesive removal test

The adhesive removal test was performed as described previously [20] to assess forepaw sensitivity and motor impairments. Briefly, an adhesive tape (0.3 × 0.3 cm) was applied to the left forepaw to cover the glabrous region. The latency to touch the tape (time to touch) and completely remove the tape (time to remove) were recorded, with a maximum of 80 s. Mice were trained for 3 days before the CCI surgery. After surgery, this test was performed for up to 35 days. Three trials were performed on each testing day and the average latencies were calculated.

#### Foot fault test

The foot fault test was performed as described previously [20] to assess sensorimotor coordination. Mice were placed on an elevated grid surface with a grid opening of 2.25 cm^2^ and videotaped for 2 min from below the grid. The videotapes were analyzed by a blinded investigator to count the number of total steps and the number of foot faults made by the left limbs (impaired side; contralateral to CCI lesion). Foot faults were recorded when the mouse misplaced its left forepaw or hindpaw such that the paw fell through the grid, and expressed as a percentage of total steps.

#### Morris water maze test

The Morris water maze test was performed as described previously [21] to assess cognitive functions. Briefly, a round platform (diameter: 11 cm) was submerged 1.5 cm under the water surface in the center of one quadrant of a circular pool (diameter: 109 cm; depth: 33 ± 0.5 cm). White tempera paint was added to the water and the temperature of the pool was maintained at 20 ± 1 °C. The test was composed of a spatial acquisition phase to assess learning capacity and a spatial retention phase to assess memory function. Spatial learning was assessed 29–33 days after CCI, where the mouse was released from one of the four quadrants and was allowed to swim for 60 s to find the hidden platform. Mice were pre-trained for 3 consecutive days before TBI (4 trials on each day). After TBI, four trails were performed on each testing day. At the end of each trial, the mouse was placed on the platform or was allowed to remain on the platform for 30 s with prominent spatial cues displayed around the room. The time spent finding the hidden platform was recorded as the latency to escape from the forced swimming task, and the mean latency of four trials was quantified as a measure of spatial learning. Spatial memory was evaluated on day 34 after CCI by removing the hidden platform. Each mouse was placed in the pool for a single 60-s probe trial. The time the mouse spent in the goal quadrant and the number of crossings the mouse made toward where the platform was previously located were recorded as spatial memory.

### Flow cytometry and fluorescence-activated single-cell sorting (FACS)

Mice were deeply anesthetized and transcardially perfused with ice-cold Hank’s balanced salt solution, and the ipsilesional and non-injured contralesional cerebral hemispheres were harvested. Single-cell suspensions were prepared from the mouse brain using a Miltenyi *Neural Tissue Dissociation Kit* and gentleMACS Octo Dissociator with Heaters according to the manufacturer’s instructions and as we described previously [22]. Suspensions were passed through a 70 μm cell strainer, and fractionated on a 30% and 70% Percoll gradient at 800×*g* for 30 min to remove myelin and cell debris. Mononuclear cells at the interface were collected, resuspended at 1 × 10^7^ cells per mL, and stained with fluorophore-conjugated antibodies (Additional file 1: Table S1). Flow cytometry was performed using a Beckman CytoFLEX flow cytometer driven by the CytExpert software. FACS was performed using a Beckman CytoFLEX SRT cell sorter driven by the Kaluza analysis software. Fluorescence compensation was performed using single-stained *OneComp eBeads* according to the manufacturer’s instructions. Data were analyzed using the FlowJo software to quantify positively stained cells.

### Real-time polymerase chain reaction (PCR)

Quantitative PCR was performed on FACS-sorted cells to verify the deletion of HDAC3. Total RNA was extracted from the sorted cells using a Qiagen *RNeasy Plus Micro Kit*, and genomic DNA was eliminated. First-strand cDNA was generated using a Qiagen *RT2 First Strand Kit* according to the manufacturer’s instructions. Real-time PCR was performed using the Qiagen *RT2 SYBR Green qPCR Mastermix* on a Roche LightCycler 480 real-time PCR system. All reactions were performed in triplicate, and the amount of mRNA was normalized to internal controls (*Gapdh*). Data are summarized as fold changes relative to WT control mice.

### Immunohistochemistry and data analyses

Mice were deeply anesthetized and transcardially perfused with 0.9% NaCl, followed by 4% paraformaldehyde in PBS. Brains were harvested and cryoprotected in 30% sucrose in PBS, and frozen serial coronal brain sections (25-μm thick) were prepared using a ThermoFisher HM525 NX cryostat. Sections were blocked with 5% donkey serum in PBS for 1 h, followed by overnight incubation (at 4 °C) with primary antibodies (Additional file 1: Table S1). For immunostaining involving mouse primary antibodies, a *Mouse on Mouse Immunodetection Kit* was used following the manufacturer’s instructions. After washing, sections were incubated for 1 h at 20 °C with donkey secondary antibodies conjugated with DyLight 488, Cy3, or Alexa Fluor 647 fluorophores (1:1000, Jackson ImmunoResearch Laboratories). Alternate sections from each experimental condition were incubated in all solutions except the primary antibodies to assess nonspecific secondary antibody staining. Sections were then mounted and coverslipped with Fluoromount-G containing DAPI (Southern Biotech). Fluorescence images were captured with a Nikon ECLIPSE Ni-E microscope, a Nikon A1 confocal microscope, or a Leica SP8 confocal microscope.

**Fig. 2:**
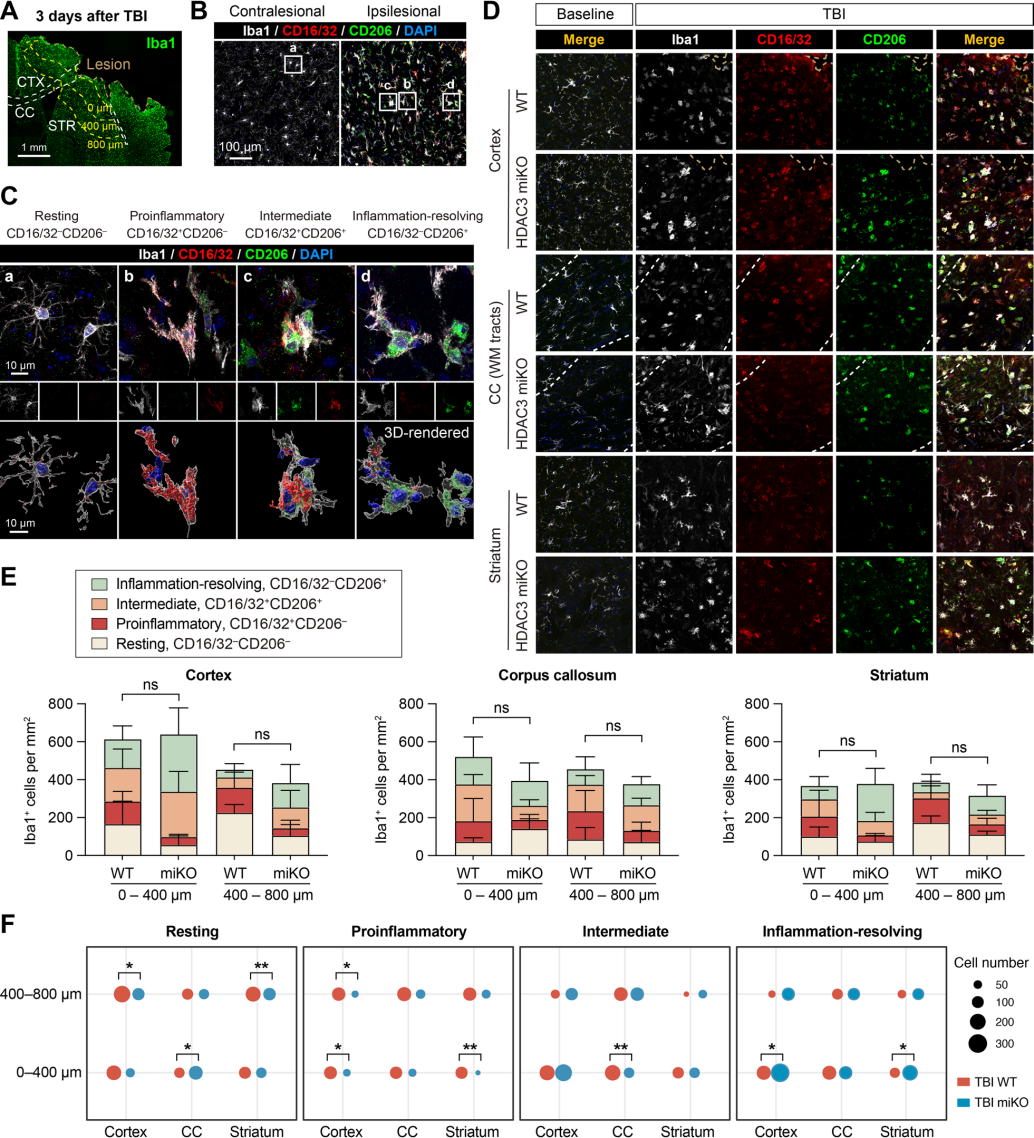
HDAC3 miKO enhances inflammation-resolving responses of microglia after TBI. HDAC3 miKO mice and WT control mice were subjected to TBI induced by controlled cortical impact. The phenotype of microglia was examined 3 days after TBI by triple-label immunostaining of CD16/32, CD206, and Iba1. **A** Iba1 immunofluorescence in the ipsilesional brain hemisphere illustrates the boundary of the TBI lesion and the peri-lesion areas in the cortex (CTX), corpus callosum (CC), and striatum (STR) where images in **B**–**D** were taken from. **B** Triple-label immunosignal of Iba1, CD16/32, and CD206 in the peri-lesion striatum and in the corresponding region in the non-injured contralesional cortex. Rectangles, areas enlarged in **C**. **C** Images taken under high magnification demonstrate 4 typical phenotypes of microglia based on their expression of CD16/32 and CD206: resting (**a**), proinflammatory (**b**), intermediate (**c**), and inflammation-resolving (**d**). Lower panels: images 3D-rendered by Imaris. **D** Representative images taken from the peri-lesion cortex, CC white matter (WM) tracts, and striatum 3 days after TBI or taken from the corresponding regions in baseline control brains. See Additional file 1: Fig. S2 for images of baseline controls under individual color channel. **E** The number of Iba1^+^ cells under each of the four phenotypic categories was counted in the peri-lesion cortex, CC and striatum, 0–400 μm and 400–800 μm from the lesion boundary. *ns* no significant difference in total Iba1^+^ cells between HDAC3 miKO and WT mice. Shown are the mean ± SD. **F** The numbers of Iba1^+^ cells in the 4 phenotype categories were illustrated in dot plots, where the area of a dot represents the number of cells. *n* = 6 (WT) and 5 (HDAC3 miKO) mice. *p < 0.05, **p < 0.01 HDAC3 miKO vs. WT

Quantitative analysis of immunofluorescence staining images was performed manually by a blinded investigator using ImageJ. Brain injury after TBI was measured on six equally spaced coronal brain sections encompassing the brain lesion immunostained for NeuN. Tissue loss was calculated as the volume of the NeuN-immunopositive contralesional hemisphere minus that of the ipsilesional hemisphere, and was expressed as a percentage of contralesional hemisphere volume. The number of resting microglia in the homeostatic brain was manually counted from 2–3 regions of interest (ROIs) per brain. Phenotypic analysis of activated microglia in the post-TBI brain was performed in the peri-lesion cortex, striatum, and corpus callosum (CC) white matter tracts. ROIs were drawn on 20× microscopic images to fully cover the proximal (0–400 μm) and distal (400–800 μm) regions surrounding the lesion site (see Fig. 2A). Positively stained cells were then counted in these regions and divided by the area of ROIs in the cortex, striatum and CC white matter tracts. Axonal injury was assessed in the CC white matter tracts and the white matter-enriched striatum in brain sections double-immunostained for β-amyloid precursor protein (β-APP) and neurofilament 200 (NF200). A threshold was set to differentiate the target signal from background. To measure β-APP and NF200-immunopositive areas, ROIs were drawn in 20× microscopic fields to outline the CC. Pixels positive for β-APP or NF200 were then measured as percentage of the total area of the ROIs. NF200^+^ area was further normalized and expressed as percentage of NF200^+^ area in the WT baseline group. Myelination was assessed in the cortex based on double-immunostaining of myelin basic protein (MBP) and NF200. Pixels immunopositive for both MBP and NF200 were considered myelinated axons, counted, and expressed as percentage of total NF200-immunopositive area. Myelination in post-TBI brains was further normalized to baseline controls.

The image-processing software Imaris was used to reconstruct three-dimensional images of Iba1, CD16/32, and CD206 immunofluorescence and quantify morphological parameters of cells as we previously described [23]. Briefly, a z-stack of images were imported into Imaris, and the immunosignal of each channel was remodeled to 3D images by the *surface* operation. Smoothing was set at 0.6 μm for all channels and images. A threshold was set to differentiate the target signal from background. Non-specific signals were manually removed, and the 3D-rendered images were constructed. All images were processed with the same adjustments. Morphological analysis of cortical microglia was performed on 3 randomly selected ROIs per brain. The parameters were automatically calculated by Imaris, including cell volume, surface area, and sphericity. We also modeled an object-oriented minimum bounding box (Bounding Box OO; Fig. 1G) for each microglia, whereby its Length A and Length C represented the length of the shortest and longest principal axes inside of the cell, respectively.

### Transmission electron microscopy (TEM)

TEM was performed as described previously [24] to assess the integrity of myelin and axons in the CC. Mice were perfused with ice-cold saline, and the harvested brains were post-fixed with 2.5% glutaraldehyde in 0.1 mol/L PBS at 4 °C. The CC tissue near the lesion site was microdissected into 1-mm^3^ blocks. The tissue was then rinsed in PBS and fixed in 1% osmium tetroxide in 0.1 mol/L PBS for 2 h. Samples were dehydrated in increasing concentrations of acetone and were embedded in Araldite resin. Ultrathin 50-nm sections were cut on a Leica UCT ultramicrotome with a DiATOME diamond knife, stained with uranyl acetate and lead citrate, and viewed using a Philips CM120 transmission electron microscope. Images were acquired in randomly selected areas within the CC from each section at a magnification of 50,000× (area per field of view: 200 μm^2^), and 3–5 images per animal were analyzed using Image J. Demyelination was identified when an axon had normal mitochondria and cytoskeletal structure but lacked myelin sheath [25]. Myelinated axons with abnormal myelin morphology including extended myelin outfold, myelin splitting, and vacuolization were counted as described previously [26], and expressed as percentages of all myelinated axons. In myelinated axons without visible morphological abnormality, the g-ratio was measured as a parameter for myelin injury using the *GRatio* plugin [27]. For each animal, 100 to 150 axons were analyzed by tracing the axonal circumference and the whole fiber circumference in a blinded fashion. The g-ratios were calculated as the ratio of the inner axonal diameter to the total outer diameter (axonal diameter + total myelin sheath thickness).

### Proteomic array analysis

Mice were deeply anesthetized and transcardially perfused with 0.9% NaCl, and fresh brain tissues were harvested. Protein was extracted from the right cerebral hemisphere using cell lysis buffer with protease and phosphatase inhibitor cocktail. The concentration of protein was measured using the Bradford protein assay. The content of 40 inflammatory factors was measured using a RayBiotech *Mouse Inflammation Array* kit following manufacturer’s instructions. Briefly, 250 µg of protein was loaded to the membrane for each sample. The membrane was incubated with biotinylated antibody cocktail at 4 °C overnight, followed by incubation with HRP-streptavidin at 4 °C overnight. The membrane was then incubated with Detection Buffer mixture and processed for chemiluminescence detection. The signal intensity for each antigen-specific antibody spot was measured using ImageJ, and background signal averaged from 3 blank spots was subtracted. The signal intensity for each inflammatory factor was then normalized to the average intensity of the 3 positive control spots on each membrane. The concentrations of various factors were expressed as fold changes relative to non-injured baseline controls.

### Compound action potential (CAP) measurements

CAPs in the CC and external capsule (EC) were measured as we described previously [28]. Briefly, fresh brains were rapidly harvested from euthanized mice, and 350-µm thick coronal brain slices were cut − 1.06 mm posterior from bregma using a Leica VT1200 S vibratome. Slices were placed in pre-gassed (95% O_2_/5% CO_2_) artificial cerebrospinal fluid (aCSF; NaCl 130 mmol/L, KCl 3.5 mmol/L, Na_2_HPO_4_ 1.25 mmol/L, MgSO_4_ 1.5 mmol/L, CaCl_2_ 2 mmol/L, NaHCO_3_ 24 mmol/L, glucose 10 mmol/L; pH 7.4) for 1 h at 20 °C, followed by incubation in a recording chamber where they were submerged and perfused at 3–4 mL/min with aCSF at 20 °C. A concentric bipolar electrode was placed into the CC approximately 0.9 mm lateral to the midline. A glass extracellular recording pipette (5–8 MΩ tip resistance when filled with aCSF) was placed into the EC, 0.75 mm and 1 mm from the stimulating electrode. The input stimuli ranged from 0 to 2 mA (100-µs duration; delivered at 0.05 Hz). The evoked CAPs were recorded by a Molecular Devices Axoclamp 700B amplifier and analyzed with pCLAMP 10 software. The average waveforms of four successive sweeps were quantified. The recording shows two positive peaks and two negative peaks conventionally referred to as N1 and N2, reflecting the responses from myelinated and unmyelinated fibers, respectively [4].

### Statistical analyses

Data are presented as mean ± standard deviation (SD). Individual data points are plotted where applicable. Statistical comparison between two groups was accomplished by the Student’s *t*-test (for normally distributed data) or Mann–Whitney *U* test (for non-normally distributed data). Differences in means among multiple groups were analyzed by one or two-way ANOVA followed by the Bonferroni/Dunn post hoc correction. A *p* value less than 0.05 was deemed statistically significant, and all testing was two-tailed. All statistics are summarized in Additional file 1: Table S4.

## Results

### Construction and characterization of microglia-specific HDAC3 knockout mice

HDAC3 is required for early embryonic development [15]. Therefore, we engineered HDAC3^LoxP^ mice with a conditional null allele of *Hdac3*, which allow temporally controlled and cell-specific deletion of HDAC3 in vivo (Fig. 1A, B). After crossing the HDAC3^LoxP^ mice with CX3CR1^CreER^ mice, we obtained tamoxifen-inducible, microglia-specific HDAC3 knockout (HDAC3 miKO) mice. Since CX3CR1 is expressed by microglia and peripheral monocytes [17], and both cells are present in the brain after TBI [29], we employed a tamoxifen pulse knockout strategy to restrict CX3CR1^CreER^-mediated HDAC3 knockout to microglia [17]. To this end, tamoxifen was first injected to HDAC3 miKO mice (75 mg/kg daily) for 5 days to induce HDAC3 deletion in all CX3CR1^+^ cells. In the next 30 days, peripheral myeloid cells would be fully replenished from CX3CR1^–^ progenitors in the bone marrow that had not undergone recombination (Fig. 1C), whereas CNS microglia had a substantially lower turnover rate [17, 30]. Therefore, only CNS microglia would retain this genomic modification 30 days after tamoxifen treatment (Fig. 1C). To verify successful and cell-specific deletion of HDAC3, we purified microglia, other brain cells and blood monocytes using FACS 30 days after tamoxifen treatment (Fig. 1D), and performed quantitative PCR on these cells. As expected, *Hdac3* mRNA was reduced by 85.8% in microglia but not in other brain cells (1.15 ± 0.20 fold of WT control; Fig. 1E). *Hdac3* mRNA was also largely recovered to baseline level in blood monocytes (84.1% ± 10.6% of WT mice) 30 days after tamoxifen treatments (Fig. 1E), demonstrating deletion of HDAC3 in microglia but not in peripheral myeloid cells.

The HDAC3 miKO mice did not exhibit any gross physical or behavioral abnormalities. We also examined the number and morphology of microglia in non-injured homeostatic brains of HDAC3 miKO mice and WT control mice using Iba1 immunostaining (Fig. 1F). HDAC3 miKO caused a 26% reduction of total microglia in the brain, but did not change the cell volume, surface area, and the shortest and longest principal axes of microglia as shown in the object-oriented minimum bounding boxes (Fig. 1G). HDAC3 miKO microglia also had slightly increased sphericity compared to WT microglia (0.32 ± 0.03 vs. 0.27 ± 0.03; Fig. 1G). To examine whether such changes in microglia altered baseline inflammation level in the brain, we measured the content of 40 inflammatory cytokines in the brain using an antibody array (Additional file 1: Fig. S1). None of the 40 inflammation markers showed significantly different expression between HDAC3 miKO and WT mice (Additional file 1: Fig. S1), suggesting that HDAC3 miKO did not change the baseline inflammation level in the non-injured homeostatic brain. In all subsequent experiments, TBI was induced in HDAC3 miKO and WT control mice 30 days after tamoxifen treatment.

### HDAC3 deficiency enhances inflammation-resolving microglial responses after TBI

To investigate the role of HDAC3 in microglial responses after TBI, we subjected adult male HDAC3 miKO mice and age- and sex-matched control WT mice to CCI, a model of moderate to severe TBI. TBI potently activated microglia, manifested by the elevation of Iba1 immunosignal in the peri-lesion areas 3 days after TBI compared to the non-injured contralesional side, including the peri-lesion cerebral cortex, the white matter tracts in the corpus callosum (CC), and the white matter-enriched striatum (Fig. 2A, B). Not only did activated microglia upregulate Iba1 after TBI, but they also expressed markers indicative of proinflammatory or inflammation-resolving phenotypes, e.g., CD16/32 and CD206 [31], which were barely detectable in quiescent microglia in the contralesional brain hemisphere (Fig. 2B) or in the non-injured baseline control brains (Fig. 2D and Additional file 1: Fig. S2). Based on their expression of the proinflammatory marker CD16/32 and the inflammation-resolving marker CD206, we classified all Iba1-immunopositive cells into four phenotypes (Fig. 2C): (1) resting/quiescent (expressing neither CD16/32 nor CD206); (2) proinflammatory (expressing CD16/32 but not CD206); (3) intermediate (expressing both CD16/32 and CD206); and (4) inflammation-resolving (expressing CD206 but not CD16/32). We then quantified the number of microglia under each phenotypic category in the peri-lesion cortex, CC and striatum in brain sections triple-immunostained with Iba1, CD16/32 and CD206 (Fig. 2D). Cell counting was performed from the proximal (0–400 μm from the lesion boundary) and distal (400–800 μm from the lesion boundary) areas in the cortex, CC and striatum (Fig. 2E). HDAC3 miKO did not alter the number of total Iba1^+^ cells in all regions examined, however, the phenotypic composition of Iba1^+^ cells was different between HDAC3 miKO mice and WT mice in these regions and at different distances from the injury site (Fig. 2E). The number of proinflammatory (CD16/32^+^ CD206^–^) microglia was dramatically reduced in HDAC3 miKO mice compared to control WT mice in the proximal areas (0–400 μm) in the cortex and striatum, with a concomitant increase of inflammation-resolving (CD16/32^–^ CD206^+^) microglia in these areas (Fig. 2F). A similar pattern was observed in the distal areas (400–800 μm) that HDAC3 miKO mice had less proinflammatory microglia and more inflammation-resolving microglia than WT mice, although the differences were less prominent than those in the proximal areas (Fig. 2F). Changes in the numbers of resting (CD16/32^–^ CD206^–^) and intermediate (CD16/32^+^ CD206^+^) microglia between HDAC3 miKO mice and WT mice demonstrated mixed patterns in the proximal and distal areas 3 days after TBI (Fig. 2E, F), indicating diverse responses in various regions and at different distances from the lesion site. In the white matter tracts in CC, fewer proinflammatory microglia and more inflammation-resolving microglia were also observed, although the changes did not reach statistical significance. Furthermore, microglia in the CC were generally less activated in HDAC3 miKO mice than WT mice, reflected by an increase of “resting microglia” and a decrease of microglia expressing both CD16/32 and CD206 (Fig. 2F). In summary, HDAC3 miKO reduced proinflammatory microglia but increased inflammation-resolving microglia without altering their total numbers, suggesting that HDAC3 miKO induces a potentially beneficial phenotypic shift of microglia at the subacute stage after TBI.

### HDAC3 miKO mitigates brain inflammation after TBI without changing the infiltration of peripheral immune cells

**Fig. 3:**
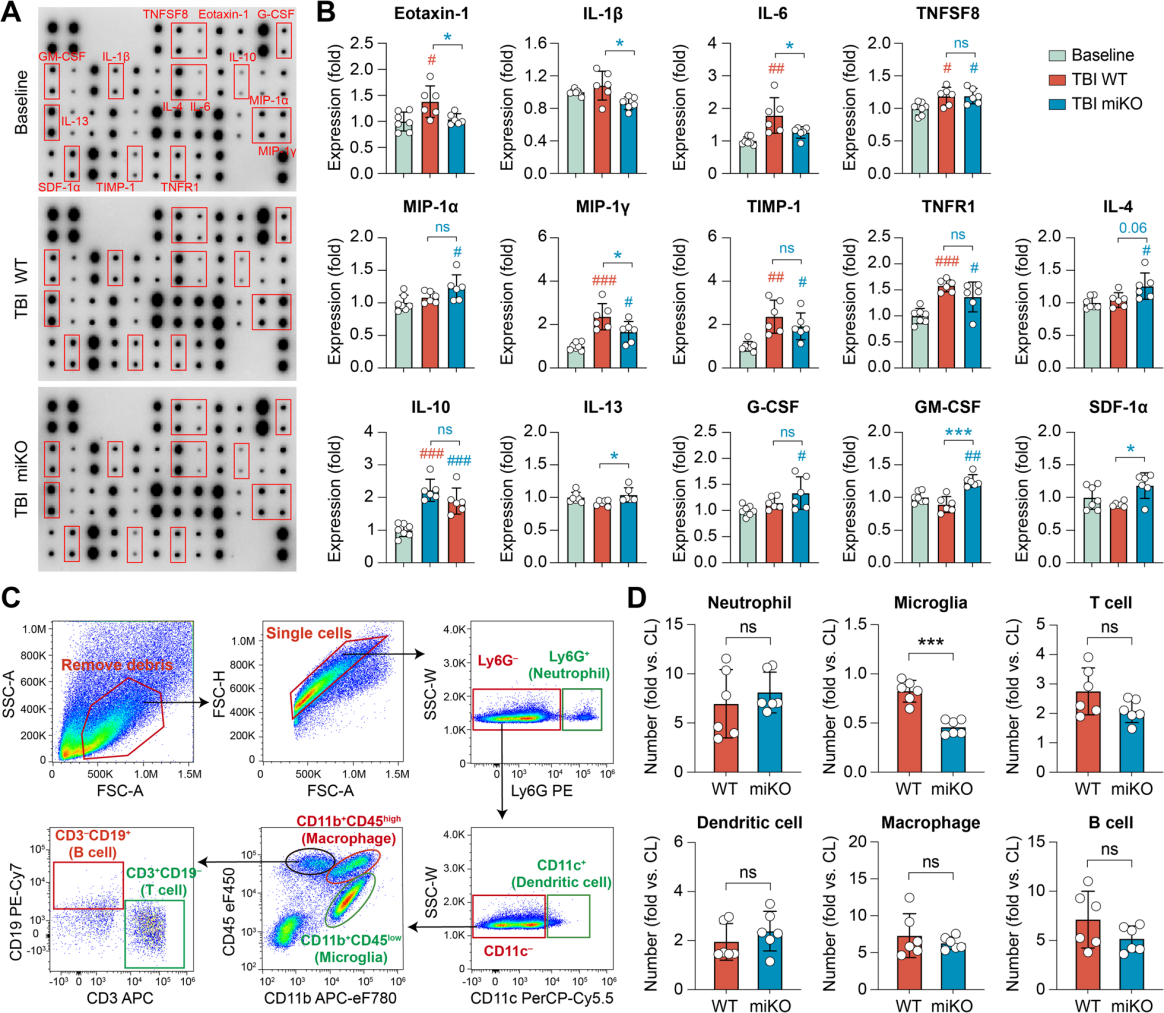
HDAC3 miKO alleviates neuroinflammation after TBI without altering infiltrating blood immune cells. HDAC3 miKO mice and WT control mice were subjected to TBI induced by CCI, and brain inflammation profiles were examined 5 days after TBI. **A**, **B** A panel of 40 inflammatory cytokines was measured in the ipsilesional brain hemisphere using an antibody array. **A** Representative blots with significantly altered markers labeled. **B** Summarized data showing the mean expression levels of 14 markers that were significantly different among groups. Non-injured baseline controls were pooled from both WT and HDAC3 miKO mice, between which there was no significant difference (see Additional file 1: Fig. S1). **C**, **D** Infiltration of peripheral immune cells into the post-TBI brain was assessed using flow cytometry 5 days after TBI. **C** Flow cytometry gating strategy for various immune cells in the brain. **D** Summarized data showing the numbers of immune cells in the ipsilesional brain hemisphere, expressed as fold changes over the non-injured contralesional side. Shown are the mean ± SD. *n* = 6–7 mice per group. ^#^*p* < 0.05, ^##^*p* < 0.01, ^###^*p* < 0.001 TBI vs. baseline control. *p < 0.05, ***p < 0.001 HDAC3 miKO vs. WT after TBI. *ns* no significant difference

Microglia are important mediators of inflammatory responses in the post-TBI brain, which critically influences TBI outcomes [32, 33]. To test whether HDAC3 miKO affects the brain inflammation milieu through modulating the phenotype of microglia, we measured the content of 40 inflammatory cytokines in the brain 5 days after TBI using an antibody array (Fig. 3A). Compared to the baseline inflammation level in the non-injured brain, TBI triggered upregulation of 11 inflammation markers in WT mice and/or HDAC3 miKO mice (Additional file 1: Fig. S3). Among these markers were cytokines that are typically proinflammatory (e.g., IL-6, TNFSF8) and chemotactic cytokines that could attract migrating immune cells (e.g., MIP-1α/CCL3, MIP-1γ/CCL9; Fig. 3B), both of which may exacerbate brain injury by potentiating neuroinflammation [34]. On the other hand, several anti-inflammatory cytokines (e.g., IL-4, IL-10) and trophic factors (e.g., G-CSF, GM-CSF) were also changed (Fig. 3B), which may be beneficial to the post-TBI brain through battling excessive inflammation and facilitating brain remodeling. We consistently observed reduced production of proinflammatory cytokines and chemokines in the brain of HDAC3 miKO mice after TBI (Fig. 3B), either that there was statistically significant difference compared to post-TBI WT mice (e.g., eotaxin-1/CCL11, IL-6), or that the levels of markers became statistically less significant when compared to baseline controls (e.g., TIMP-1, TNFR1). Furthermore, we observed a trend of HDAC3 miKO mice upregulating several anti-inflammatory cytokines or trophic factors (Fig. 3B), such as IL-4 (*p* = 0.01 vs. baseline), IL-13 (*p* < 0.05 vs. TBI WT), G-CSF (*p* = 0.02 vs. baseline), GM-CSF (*p* < 0.0001 vs. TBI WT), and SDF-1α (*p* = 0.02 vs. TBI WT), suggesting that HDAC3 miKO may facilitate the resolution of neuroinflammation and brain remodeling/repair after TBI.

Post-TBI brain inflammation is contributed by not only resident microglia, but also peripheral immune cells that invade the brain through the damaged blood–brain barrier. Therefore, we sought to examine whether HDAC3 miKO impacted the responses of infiltrating peripheral immune cells in the post-TBI brain. Using flow cytometry (Fig. 3C), we detected a strong increase of various immune cells in the post-TBI brain compared to the non-injured contralesional hemisphere, including neutrophils (6.96 ± 3.47 fold vs. contralesional), dendritic cells (1.95 ± 0.74 fold vs. contralesional), macrophages, and lymphocytes (Fig. 3D). TBI-induced changes in all these immune cells were comparable between WT mice and HDAC3 miKO mice 5 days after TBI (Fig. 3D), suggesting that HDAC3 miKO did not induce major changes in the infiltrating behavior of peripheral immune cells. Interestingly, the “microglia” population (CD11b^+^CD45^low^ cells) became smaller than the contralesional hemisphere after TBI in both WT and HDAC3 miKO mice (Fig. 3D), which may in part result from microglia upregulating CD45 during activation and being classified as “macrophages” (CD11b^+^CD45^high^ cells) by flow cytometry. In summary, our data showed that HDAC3 miKO markedly mitigated brain inflammation burden after TBI without changing peripheral immune cells, suggesting that HDAC3 miKO-afforded anti-inflammatory effects were primarily due to its modulation on the phenotype of microglia.

### HDAC3 miKO is sufficient to improve long-term functional recovery after TBI

**Fig. 4:**
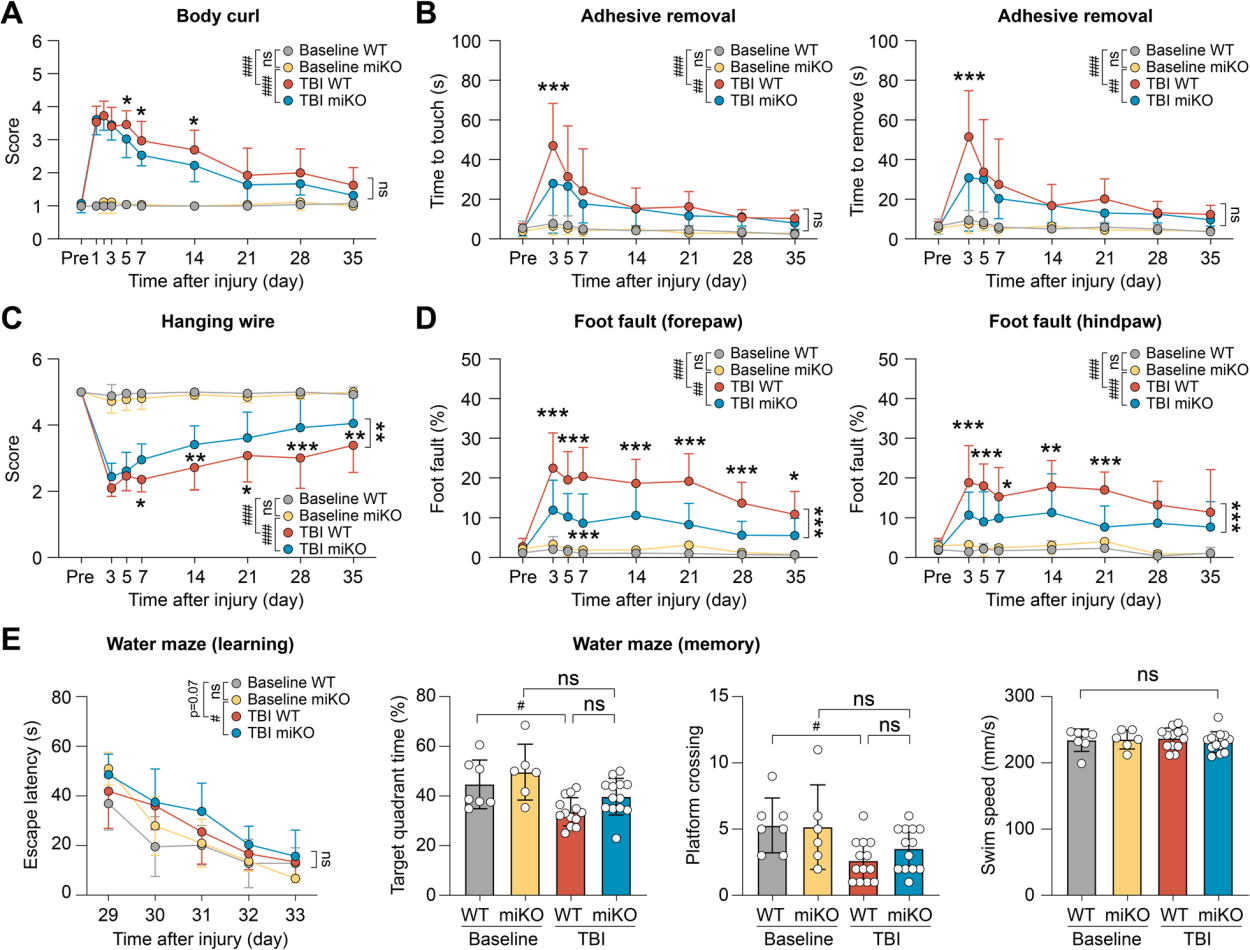
HDAC3 miKO promotes long-term functional recovery after TBI. Adult male HDAC3 miKO mice and age- and sex-matched WT control mice were subjected to TBI induced by CCI or non-injury control procedures (baseline control). **A**–**D** Sensorimotor deficits were assessed in male mice before (Pre) and up to 35 days after injury by the body curl (**A**), adhesive removal (**B**), hanging wire (**C**), and foot fault (**D**) tests. *n* = 9 (baseline) or 13 (TBI) mice per group. **E** The Morris water maze test was performed to assess spatial learning and spatial memory in male mice at 29–34 days after injury. Mice in all groups had comparable swim speeds, suggesting similar gross locomotor functions. *n* = 6–7 (baseline) or 13 (TBI) mice per group. Shown are the mean ± SD. ^#^*p* < 0.05, ^##^*p* < 0.01, ^###^*p* < 0.001 TBI vs. baseline. *p < 0.05, **p < 0.01, ***p < 0.001 HDAC3 miKO vs. WT after TBI. *ns* no significant difference

How important are HDAC3 miKO-induced changes in microglial phenotype in impacting the overall outcomes after TBI? To address this question, we evaluated the recovery of neurological functions in HDAC3 miKO mice after TBI over a period of 35 days. We used a panel of four behavioral tests (body curl, adhesive removal, hanging wire and foot fault tests) to examine the sensorimotor functions of the adult male mice (Fig. 4A–D). All four tests detected remarkable sensorimotor deficits in mice for at least 35 days after TBI compared to baseline control mice without injury (Fig. 4A–D). Compared to WT mice, HDAC3 miKO mice performed significantly better at 5, 7, and 14 days after TBI in the body curl test (Fig. 4A; *p* < 0.05) and at 3 days after TBI in the adhesive removal test (Fig. 4B; *p* < 0.001). However, HDAC3 miKO mice did not demonstrate statistically significant improvement compared to WT mice over the entire testing course (*p* > 0.05 between HDAC3 miKO mice and WT mice by two-way repeated measures ANOVA). On the other hand, in the hanging wire test (Fig. 4C) and foot fault test (Fig. 4D), HDAC3 miKO mice performed significantly better than WT mice during the entire testing period of 35 days after TBI. At 35 days after TBI, the performance of WT mice was at 68.7% of the performance of baseline control mice in the hanging wire test, whereas HDAC3 miKO mice recovered to 81.0% of baseline levels (Fig. 4C). Consistently, forepaw and hindpaw foot faults in HDAC3 miKO mice were only 51.2% and 67.8%, respectively, of the foot faults made by WT mice at 35 days after TBI (Fig. 4D), suggesting that HDAC3 miKO mice had improved recovery of sensorimotor functions after TBI. We also performed the Morris water maze test to assess the animals’ spatial learning and spatial memory, but HDAC3 miKO did not elicit significant improvement compared to WT mice in either the learning phase or the memory phase of this test at 29–34 days after TBI (Fig. 4E). Together, these data suggest that by promoting anti-inflammatory and pro-resolving microglial responses, HDAC3 miKO is sufficient to elicit long-term improvement of functional recovery after TBI.

### Deletion of microglial HDAC3 promotes white matter integrity after TBI

**Fig. 5:**
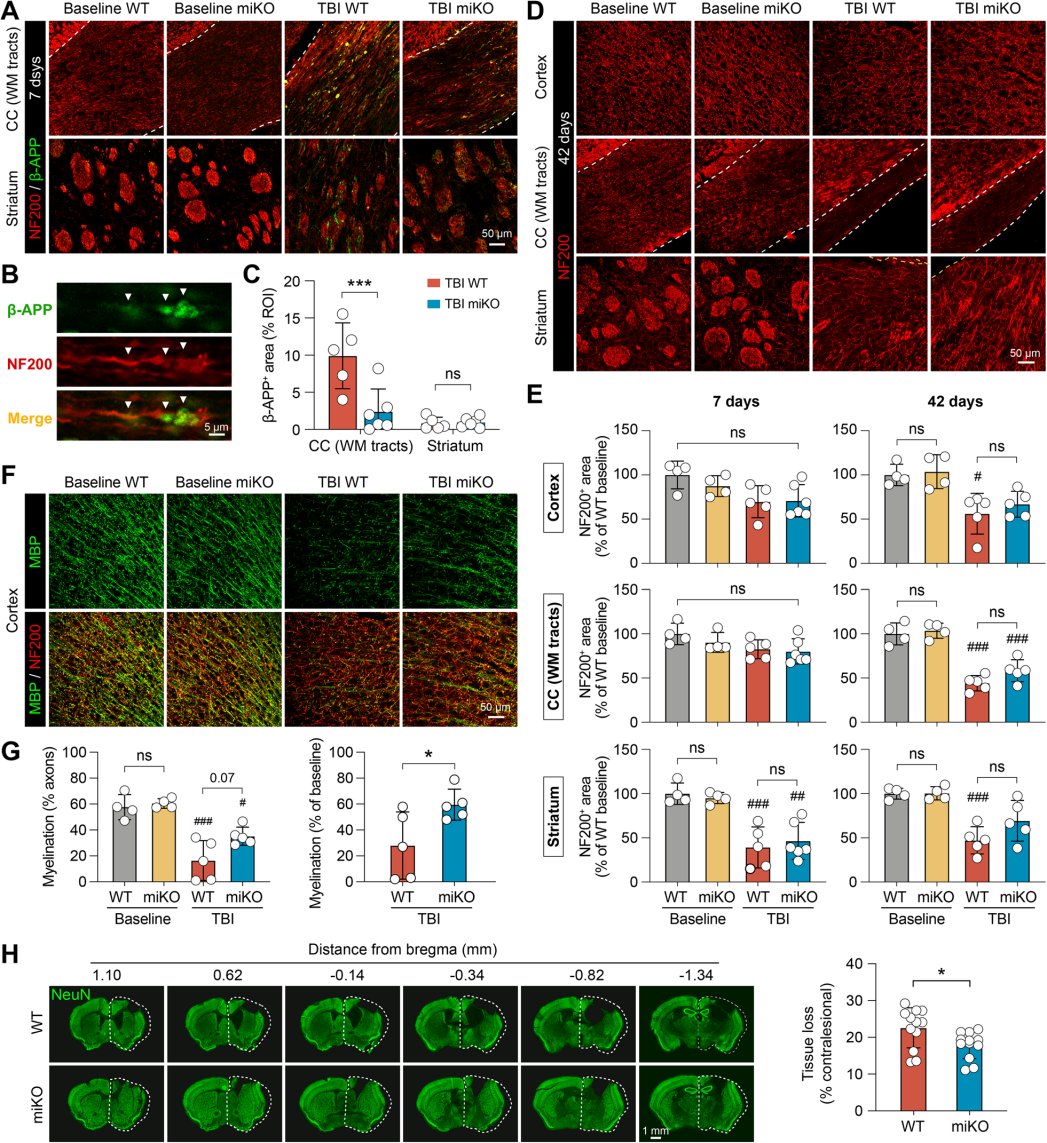
HDAC3 miKO improves short-term and long-term integrity of white matter after TBI. Immunofluorescence staining was performed at 7 and 42 days after TBI or non-injury control procedures (baseline controls) to assess brain injury and white matter integrity in HDAC3 miKO and WT mice. **A**–**C** Axonal injury was assessed 7 days after TBI using NF200 and β-APP double-label immunostaining. **A** Representative images taken from the white matter-enriched corpus callosum (CC) and striatum in the ipsilesional brain hemisphere. Dashed line, the boundary of CC. **B** β-APP immunosignal in the shape of classic axonal bulbs and varicosities (arrows) was observed after TBI, suggesting axonal damage. **C** Summarized data on β-APP-immunopositive areas. **D** Axonal integrity was assessed 42 days after TBI by NF200 immunostaining. **E** Summarized data on NF200-immunopositive areas in the ipsilesional cortex, CC and striatum 7 and 42 days after TBI, expressed as percentages of the WT baseline group. **F** Myelin integrity in the peri-lesion cortex was assessed 42 days after TBI using MBP and NF200 double-immunostaining. **G** Summarized data on the degree of myelination (areas immunopositive for both MBP and NF200), expressed as percentages of myelinated axons to total axons (left panel), or as percentages to baseline controls (right panel). *n* = 4 (baseline) or 5–6 (TBI) per group. **H** The volume of chronic brain tissue loss was measured 42 days after TBI on coronal brain sections immunostained for the neuronal marker NeuN. Dashed line, the relative area of the contralesional hemisphere to illustrate ipsilesional tissue loss. *n* = 12 mice per group. Shown are the mean ± SD. ^#^*p* < 0.05, ^##^*p* < 0.01, ^###^*p* < 0.001 TBI vs. baseline. *p < 0.05, ***p < 0.001 HDAC3 miKO vs. WT. *ns* no significant difference

Given the sustained superior performance of HDAC3 miKO mice in various neurobehavioral tests compared to WT control mice after TBI, we speculated that brain injury was ameliorated in HDAC3 miKO mice as a result of the phenotypic switch of microglia toward an inflammation-resolving phenotype. We therefore harvested the brains of HDAC3 miKO mice and WT mice at 7 days and 42 days after TBI to examine brain injury at the subacute and chronic stages, respectively. First, we assessed TBI-induced axonal damage 7 days after TBI using double-label immunostaining of the axonal marker NF200 and β-APP, a marker for axonal injury [28] (Fig. 5A). We observed prominent accumulation of β-ΑPP after TBI in the peri-lesion CC and, to a less extent, in the striatum (Fig. 5A, B), reflecting axonal damage. We found that the level of β-APP was markedly lower in HDAC3 miKO mice than WT mice 7 days after TBI (Fig. 5A, C). On the other hand, disruption of axonal integrity was less robust as detected by NF200 immunostaining 7 days after TBI, whereby a reduction of NF200 immunosignal was observed only in the striatum (Fig. 5E). Such injury deteriorated, however, in the next 5 weeks, and NF200 immunosignal was significantly lower in the ipsilesional cortex, CC and striatum in WT mice 42 days after TBI than in baseline controls (Fig. 5D, E). HDAC3 mKO mice had larger average NF200-immunopositive areas in these regions than WT mice 42 days after TBI, suggesting improved long-term axonal integrity (Fig. 5D). Although we did not find statistically significant differences in NF200^+^ areas between HDAC3 miKO mice and WT mice, TBI-induced loss of NF200 expression was no longer significantly different from baseline controls in HDAC3 miKO mice 42 days after TBI in the peri-lesion cortex and striatum (Fig. 5E).

We also assessed the integrity of myelin, another major component of the white matter, in the post-TBI cerebral cortex using immunostaining of myelin basic protein (MBP; Fig. 5F). We identified myelinated axons as areas immunopositive for both MBP and NF200, and observed a 72.0% reduction of axonal myelination in WT mice 42 days after TBI compared to baseline controls (Fig. 5G), indicating disruption of myelin integrity after TBI. Although myelination in HDAC3 miKO mice 42 days after TBI remained significantly less than in baseline control mice (Fig. 5G left panel), such demyelination in HDAC3 miKO mice was markedly alleviated compared to WT mice (Fig. 5G right panel; 59.6% vs. 28% of baseline controls, *p* < 0.05). At 42 days after TBI, the gross lesion volume was also significantly smaller in HDAC3 miKO mice than WT mice, as calculated from serial coronal brain sections immunostained for the neuronal marker NeuN (Fig. 5H). Together, these data demonstrated that HDAC3 miKO reduced brain injury after TBI and, in particular, promoted the integrity of axons and myelin in the white matter.

### HDAC3 miKO improves nerve signal conduction in the white matter after TBI

**Fig. 6:**
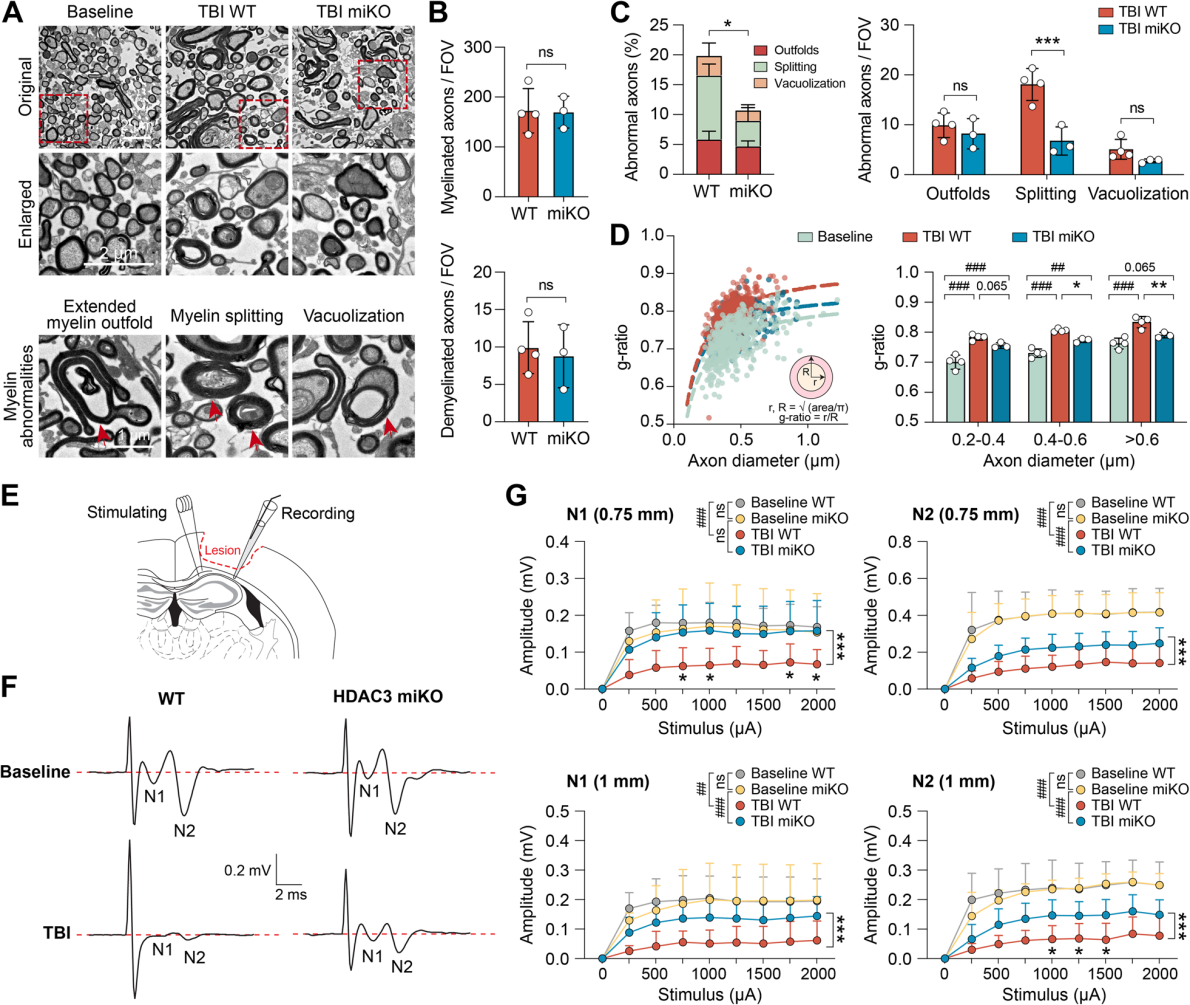
HDAC3 miKO improves nerve signal conduction in the white matter after TBI. **A**–**D** White matter ultrastructure was examined in the ipsilesional CC 42 days after TBI using transmission electron microscopy (TEM). **A** Representative TEM images. TBI induced demyelination and abnormal myelin morphology in myelinated axons (lower panel), such as extended myelin outfold, myelin splitting, and vacuolization (arrow). **B**, **C** The numbers of myelinated axons, demyelinated axons, and myelinated axons with abnormal myelin morphology were counted in post-TBI HDAC3 miKO mice and control WT mice. Data were expressed as the number of axons per field of view (FOV; 200 μm^2^) or percentage of total myelinated axons. **D** Scatter plot (left panel) and summarized data (right panel) showing the g-ratio of all axons counted from 3 to 4 mice in each group. Baseline control group was pooled from both WT and HDAC3 miKO mice. **E**–**G** Axonal conduction at the ipsilesional CC/external capsule was assessed 42 days after TBI by measuring the evoked compound action potentials (CAPs). **E** Diagram showing the location of the stimulating and recording electrodes at the external capsule. Dashed line: the boundary of the lesion. **F** Representative traces of the evoked CAPs at 2000 µA stimulation to show the N1 (through myelinated axons) and N2 (through unmyelinated axons) components, recorded 1 mm from the stimulation point. **G** Quantification of the N1 and N2 amplitudes after various stimulation current intensities, recorded 0.75 mm and 1 mm from the stimulating point. *n* = 5–8 mice per group. Shown are the mean ± SD. ^##^*p* < 0.01, ^###^*p* < 0.001 TBI vs. baseline. *p < 0.05, ***p < 0.001 HDAC3 miKO vs. WT after TBI. *ns* no significant difference

We further performed transmission electron microscopy to confirm the beneficial effects of HDAC3 miKO on myelin and axonal integrity after TBI (Fig. 6A). Ultrastructural examination identified demyelinated axons and myelinated axons with overtly abnormal myelin morphology in the post-TBI CC (Fig. 6A), such as extended myelin outfolds, myelin splitting, and vacuolization [26]. Quantitative analyses showed that the total numbers of demyelinated and myelinated axons were comparable between HDAC3 miKO mice and WT mice (Fig. 6B). However, myelinated axons with abnormal myelin morphology were significantly fewer in HDAC3 miKO mice than in WT mice 42 days after TBI (Fig. 6C). Among all 3 types of myelin abnormalities, myelin splitting was especially ameliorated in HDAC3 miKO mice (Fig. 6C). In myelinated axons with no evident aberration, we calculated the g-ratio—a commonly used parameter to assess the degree of demyelination. Consistently, the g-ratio was also smaller in HDAC3 miKO mice than WT mice 42 days after TBI (Fig. 6D), reflecting ameliorated demyelination in HDAC3 miKO mice.

White matter plays a fundamental role in facilitating signal transmission between different brain regions. Would the improved axonal and myelin integrity in HDAC3 miKO mice lead to better nerve signal conduction after TBI? To this end, we measured the evoked compound action potentials (CAPs) in the CC 42 days after TBI (Fig. 6E) as an indicator of white matter functionality [35]. The CAPs typically consist of an initial “N1” wave mediated primarily by faster-conducting myelinated axons (and thus has larger velocity), followed by a slower “N2” wave mediated by unmyelinated axons [35] (Fig. 6F). As expected and consistent with our previous reports [4, 28], the amplitude of both N1 and N2 segments was notably reduced after TBI, suggesting injury and/or functional depression of myelinated and unmyelinated nerve fibers (Fig. 6F, G). Importantly, both the N1 and N2 components of CAPs had larger amplitudes in HDAC3 miKO mice than in WT mice 42 days after TBI, as measured from 2 distances (0.75 mm and 1 mm from the stimulating point; Fig. 6F, G), suggesting that HDAC3 miKO ameliorated the functional depression of both myelinated axons (the N1 “fast” component) and unmyelinated axons (the N2 “slow” component) after TBI. In summary, our data obtained from combined immunohistochemical, electron microscopical and electrophysiological approaches consistently demonstrated that HDAC3 miKO results in greater recovery of the structural and functional integrity of white matter after TBI.

## Discussion

In this study, we created a novel conditional knockout mouse model to achieve microglia-specific HDAC3 deletion in vivo, and revealed a significant role of HDAC3 in proinflammatory microglial responses after TBI. We further showed that by potentiating brain inflammation, microglial HDAC3 depressed long-term functional recovery after TBI and is therefore a promising therapeutic target.

HDAC activity has long been linked to worse outcome after TBI, largely for the reason that pan-HDAC inhibitors consistently demonstrate beneficial effects in reducing brain injury and promoting functional recovery after TBI [2–5]. Eukaryotic cells express a variety of HDAC isoforms, among which functional uniqueness or redundancy may prevail. Deciphering the role of individual HDACs is therefore imperative to developing inhibitors with improved targeting specificity and efficacy and with fewer side effects. Due to the pan-targeting nature of those HDAC inhibitors used in prior TBI research, it is unknown whether the targeted cluster of HDACs have redundant functions, or whether one or more isoforms play a dominant role in dictating TBI outcomes. Furthermore, HDAC inhibitors usually elicited a plethora of salutary cellular responses accompanying the reduced brain lesion, among which the primary protective action cannot be recognized. Our study aimed to evaluate the role of HDAC3, the most abundantly expressed Class I HDAC in the brain [36], with an emphasis on microglia-mediated inflammatory responses after TBI. Not only did our conditional HDAC3 knockout mice bypass the developmental requirement of HDAC3 and embryonic lethality caused by conventional global HDAC3 knockout, our model also specifically delineated microglial HDAC3 after TBI in vivo for the first time. Although we cannot preclude functional compensation from other HDACs upon the loss of HDAC3, those changes are likely to be secondary to the absence of HDAC3. Our results therefore advocate testing the efficacy of selective HDAC3 inhibitors in TBI, which would improve targeting specificity over pan-HDAC inhibitors.

Another innovative aspect of our study is the tamoxifen pulse knockout strategy that largely restricts HDAC3 deletion to microglia. The role of microglia in neurological disorders is mostly investigated together with monocyte-derived macrophages in existing studies. This is because macrophages are almost always present in the diseased or injured brain as a result of blood–brain barrier disruption, and they are virtually indistinguishable from activated microglia by commonly used markers such as CD11b and Iba1 [37]. This limitation is being tackled with the use of emerging microglia-specific markers identified by single-cell analyses such as TMEM119 [38], and CreER mice driven by the *Tmem119* promoter were recently generated [39]. Despite excellent efficiency in labeling homeostatic microglia, such microglial markers are often downregulated under disease or injury conditions [40], limiting their use to tag microglia with high fidelity in the diseased or injured brain. We utilized CX3CR1—a strong promoter active in both microglia and monocytes—and took advantage of the different turnover rates of microglia and monocytes [30] to delete HDAC3 only in microglia. As originally characterized by Goldmann et al. [30], genomic modification mediated by the CX3CR1^CreER^ mice persisted in microglia for more than 12 weeks after the initial tamoxifen treatment. Intriguingly, deletion of HDAC3 resulted in a reduction in the number of microglia in the homeostatic brain, and HDAC3-null microglia appeared to be slightly activated based on their morphology (Fig. 1F, G). We do not know the exact mechanism underlying such changes, but it could be related to blocked proliferation and/or increased apoptosis for cells deficient in HDAC3 [41, 42]. This did not seem to alter the baseline brain inflammation level, however, as the expression of common inflammatory cytokines in the brain did not differ between HDAC3 miKO mice and WT mice (Additional file 1: Fig. S1). Furthermore, the long-lasting protective effects of microglia-targeted HDAC3 deletion against TBI could not be explained by the reduction of microglia numbers, because these numbers rebounded 3 days after TBI to the levels of WT controls (Fig. 2E). Nevertheless, future studies are warranted to examine in-depth the changes occurring in the transcriptome and biology of microglia when HDAC3 is ablated. We observed that HDAC3 miKO led to phenotypic switch of microglia from proinflammatory to inflammation-resolving phenotype at the subacute stage after TBI, whereas TBI-induced infiltration of blood immune cells was unchanged in HDAC3 miKO mice, suggesting the mitigation of neuroinflammation in HDAC3 miKO mice was contributed predominantly by microglia and not by peripheral immune cells including monocyte-derived macrophages. It should be noted that there are several limitations in our current experimental design. Firstly, we characterized the proinflammatory and inflammation-resolving phenotypes of microglia using Iba1 as a cell marker, which also labels macrophages. Secondly, activated microglia upregulate CD45 and would become indistinguishable from macrophages (CD11b^+^CD45^high^ cells) in flow cytometry. This partially explained why the “microglia” population (CD11b^+^CD45^low^ cells) turned smaller after TBI compared to baseline controls. Thirdly, although TBI-induced brain infiltration of peripheral immune cells was comparable between HDAC3 miKO mice and WT mice, we cannot rule out functional differences in these cells. Notwithstanding these caveats of the present study, any alteration caused by HDAC3 miKO on peripheral immune cells would, again, be secondary to changes happening in microglia. These unanswered questions may be addressed in the future by transcriptomic profiling at the single-cell level to accurately distinguish microglia versus macrophages based on the whole-transcriptome.

We adopted a routinely used pair of markers (i.e., CD16/32 and CD206) to label proinflammatory and inflammation-resolving microglia [31], but it should be noted that the heterogeneity of microglia is far more complicated than what could be represented by several pre-selected markers. The emerging single-cell analytic tools would allow unbiased characterization of microglial subsets that does not rely on pre-defined markers in future studies. Our data suggest that lack of HDAC3 propels microglia toward an overall inflammation-resolving phenotype that benefits the brain after TBI, but the downstream molecular targets remain to be elucidated. Deacetylating the amino-terminal tails of histones, HDAC3’s canonical function is to place transcription repression through modulating chromatin architecture, whereby its deletion may increase the transcription of anti-inflammatory genes among all targeted substrates. On the other hand, HDAC3 could directly modulate the acetylation status and therefore the activity of proinflammatory transcription regulators such as STAT1 [43]. *Hdac3*^*−/−*^ bone marrow-derived macrophages underwent a cascade of changes in their inflammatory gene expression program when stimulated with lipopolysaccharides, featuring compromised interferon-β and STAT1 expression [14]. The present study focused on the pathophysiological impact of microglial HDAC3 on the overall brain inflammation burden and functional recovery in animals at the systemic level. HDAC3 thereupon may act as a hub molecule, the manipulation of which was adequate to induce changes that eventually shape the overall functional phenotype of microglia.

White matter is vulnerable to traumatic injury and its integrity is vital for nerve signal conduction and functional restoration after TBI. The phenotype of microglia/macrophages actively regulates white matter injury and repair, and a shift from anti-inflammatory to proinflammatory microglial/macrophage phenotype from subacute to chronic stages after TBI is correlated to the progressive deterioration of white matter [12]. Anti-inflammatory and immunomodulatory “M2” microglia/macrophages could also produce trophic factors that enhance white matter repair after injury [44]. The present study examined both the structure and functionality of white matter as parameters of long-term TBI outcomes upon phenotypic modulation of microglia by HDAC3 deletion. We utilized immunostaining, electron microscopy, and electrophysiology to evaluate 3 types of axonal injury: (1) robust axonal degeneration: reflected by the loss of NF200 immunosignal; (2) subtle breach of axonal integrity: reflected by the accumulation of β-APP where axons lose integrity, with or without overt reduction of NF200 immunosignal; and (3) functional depression of morphologically intact axons: reflected by the impairment of nerve fiber conduction in electrophysiological measurements. TBI-induced loss of NF200 immunosignal at 7 days and 42 days was not significantly ameliorated by HDAC3 miKO (Fig. 5E). However, accumulation of β-APP in the white matter tracts of CC at 7 days after TBI and TBI-induced demyelination were both rescued by HDAC3 miKO (Fig. 5C, G). Such protective effects of HDAC3 miKO on axons and myelin were further supported by CAP measurements, which reflected both the structure integrity and functionality of axons and myelin. Overall, our interpretation of the data is that although HDAC3 miKO does not prevent the (partial) loss of NF200-positive axons, it improves the functional (increased CAPs) and structural (decreased β-APP) integrity of the remaining axons after TBI. We believe that these protective effects were attributed to microglia, because our miKO mice specifically targeted microglia and other effects were therefore secondary to HDAC3 deletion in microglia. In the future, studies employing non-invasive techniques to assess brain injury in live animals, such as in vivo MRI and DTI which examine gray matter and white matter injuries, will enable within-subject, longitudinal assessment of injury progression and allow us to collect more parameters from the same animals for multifactor regression analyses among microglia signatures, tissue loss and neurorecovery. By alleviating destructive proinflammatory microglial responses, HDAC3 miKO could reduce axonal injury and facilitate white matter repair. This is supported by the elevation of several trophic factors in the brain of HDAC3 miKO mice after TBI (Fig. 3B). Such improved white matter integrity likely underlay the enhanced functional recovery for at least 6 weeks after TBI, the most exciting effect of HDAC3 miKO. Interestingly, we observed improved motor functions but not spatial learning and memory in HDAC3 miKO mice after TBI, suggesting disparate effects on different brain regions or anatomical pathways. However, this does not preclude HDAC3 miKO’s possible beneficial effects on other cognitive functions that do not rely on spatial cues, e.g., those assessed by the novel object recognition test [45] or some paradigms of passive avoidance test [46]. Evaluation of the animals’ performance in these additional behavioral tests is beyond the scope of the present study, but is an important focus for future studies to pinpoint the neural circuits affected by microglial modulation after TBI [47, 48]. It should also be noted that adult female HDAC3 miKO mice also performed better than age-matched female WT mice in the hanging wire test and foot fault test (preliminary observations) over a testing period of 35 days after TBI, suggesting that sex as a biological variable does not affect the beneficial effect of HDAC3 miKO in improving functional recovery after TBI.

There remains urgent need to develop effective therapies that can battle the neurological deficits after TBI. Our data show that HDAC3 is a promising therapeutic target to ameliorate excessive neuroinflammation and promote long-term functional recovery after TBI. To date, no study has tested the efficacy of selective HDAC3 inhibitors against TBI, one candidate being the *N*-(*o*-aminophenyl) carboxamide RGFP966. RGFP966 is brain-penetrant and highly specific to HDAC3 over other HDACs, and subcutaneous dosing of RGFP966 at 10 mg/kg in mice results in brain concentrations higher than the IC_50_ of RGFP66 for HDAC3 within 15 min [49]. RGFP966 was previously tested in models of spinal cord injury, in which it efficaciously modulated the function of microglia/macrophages and alleviated local inflammation [50, 51]. Whether RGFP966 could improve the functional recovery after spinal cord injury, however, is controversial in prior studies [50, 51]. While Kuboyama et al. reported that intraperitoneal RGFP966 treatment (10 mg/kg) for 2 days led to sustained improvement of functional recovery for 30 days after spinal cord injury in female mice [51], Sanchez et al. reported neutral results in functional recovery of female mice treated with the same dose of RGFP966 for 3 days [50]. Given its excellent selectivity and in vivo pharmacokinetic properties, it would be an important future research direction to evaluate whether RGFP966 can elicit sustained improvement of outcome after TBI.

## Conclusions

HDAC3 is essential to the proinflammatory microglial phenotype after TBI. Ablation of microglial HDAC3 effectively mitigates post-TBI neuroinflammation and facilitates long-term functional recovery. HDAC3 is therefore a promising therapeutic target for future TBI treatment to boost beneficial microglial responses and improve long-term outcomes.

## Supplementary Information

**Additional file 1: Figure S1.** Microglia-specific HDAC3 knockout does not alter baseline inflammation level in the homeostatic brain. **Figure S2.** Microglial expression of activation markers under baseline conditions (related to Fig. 2). **Figure S3.** HDAC3 miKO promotes the resolution of inflammation in the brain after TBI (related to Fig. 3). **Table S1.** Key resources. **Table S2.** The numbers of mice used in this study. **Table S3.** Tissue samples processed in this study. **Table S4.** Statistics reporting.

**Additional file 2.** Raw image of agarose gel in Fig. 1B.

## Data Availability

The datasets used and/or analyzed in this study are available from the corresponding author upon reasonable request.

## Abbreviations

aCSF: Artificial cerebrospinal fluid
APP: Amyloid precursor protein
CAP: Compound action potential
CC: Corpus callosum
CCI: Controlled cortical impact
EC: External capsule
ES cell: Embryonic stem cell
FACS: Fluorescence-activated cell sorting
HDAC: Histone deacetylase
MBP: Myelin basic protein
miKO: Microglia-specific knockout
NF200: Neurofilament 200
PCR: Polymerase chain reaction
ROI: Region of interest
TBI: Traumatic brain injury
TEM: Transmission electron microscopy
WT: Wild type
