# Supplementary material for: Microglia-specific deletion of histone deacetylase 3 promotes inflammation resolution, white matter integrity, and functional recovery in a mouse model of traumatic brain injury
Source: J Neuroinflammation. 2022 Aug 6;19:201. doi: 10.1186/s12974-022-02563-2 (PMC9357327; doi:10.1186/s12974-022-02563-2)
Supplement: Supplementary file 1 — Additional file 1: Figure S1. Microglia-specific HDAC3 knockout does not alter baseline inflammation level in the homeostatic brain. Figure S2. Microglial expression of activation markers under baseline conditions (related to Fig. 2). Figure S3. HDAC3 miKO promotes the resolution of inflammation in the brain after TBI (related to Fig. 3). Table S1. Key resources. Table S2. The numbers of mice used in this study. Table S3. Tissue samples processed in this study. Table S4. Statistics reporting. [file 12974_2022_2563_MOESM1_ESM.pdf]

# Additional file 1

# Microglia-specific deletion of histone deacetylase 3 promotes inflammation resolution, white matter integrity, and functional recovery in a mouse model of traumatic brain injury

Yongfang Zhao[1], Hongfeng Mu[1], Yichen Huang[1], Sicheng Li[1], Yangfan Wang[1], R. Anne Stetler[1,4], Michael V. L. Bennett[2], C. Edward Dixon[3,4], Jun Chen[1,4], and Yejie Shi[1,4]

[1]Pittsburgh Institute of Brain Disorders & Recovery and Department of Neurology, University of Pittsburgh, Pittsburgh, PA 15213, USA

[2]Dominick P. Purpura Department of Neuroscience, Albert Einstein College of Medicine, Bronx, NY 10461, USA

[3]Department of Neurosurgery, University of Pittsburgh, Pittsburgh, PA 15213, USA

[4]Geriatric Research, Education and Clinical Center, Veterans Affairs Pittsburgh Health Care System, Pittsburgh, PA 15261, USA

**Corresponding authors:**

Jun Chen (chenj2@upmc.edu)

Yejie Shi (y.shi@pitt.edu)

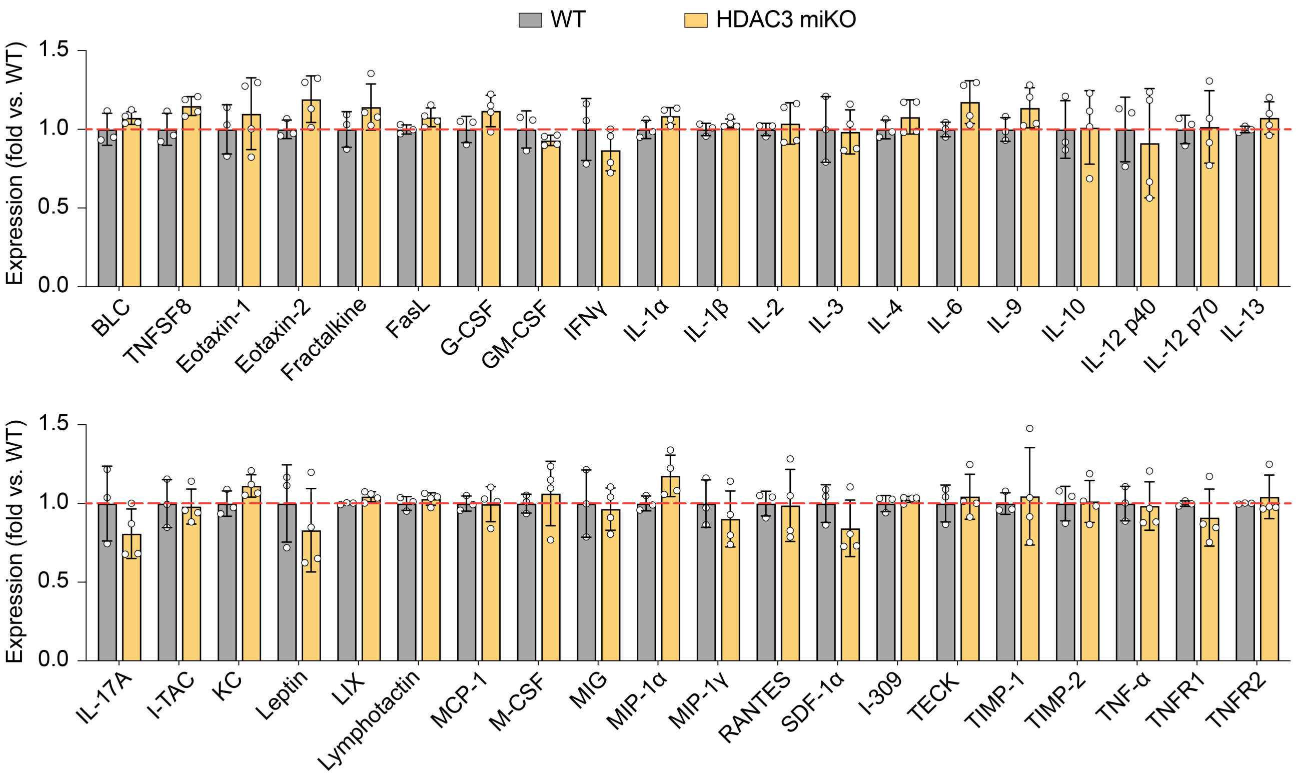


**Figure S1. Microglia-specific HDAC3 knockout does not alter baseline inflammation level in the homeostatic brain.**

HDAC3 miKO mice and WT mice received tamoxifen treatment for 5 days, followed by a 30-day latency. The content of 40 inflammatory cytokines was measured in the brain using an antibody array, and the data were expressed as fold changes versus WT mice. n=3 (WT) or 4 (HDAC3 miKO) mice per group. $p>0.05$ between HDAC3 miKO mice and WT mice for all markers.

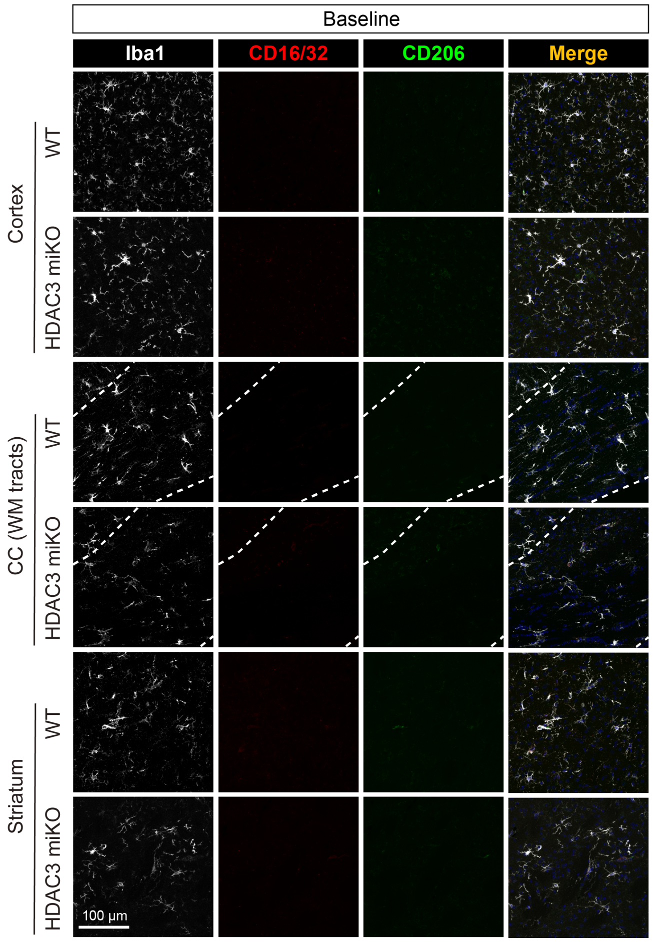


**Figure S2. Microglial expression of activation markers under baseline conditions (related to Fig. 2).**

The phenotype of microglia was examined in HDAC3 miKO mice and WT control mice under baseline conditions by triple-label immunostaining of CD16/32, CD206, and Iba1. Shown are images taken from the cortex, corpus callosum (CC) white matter (WM) tracts, and striatum, representative of 3 WT mice and 2 HDAC3 miKO mice. Dashed line, the boundary of CC. All microglia displayed morphology indicative of a resting/quiescent state, and there was no detectable immunosignal of the microglia activation marker CD16/32 or CD206 in the regions examined.

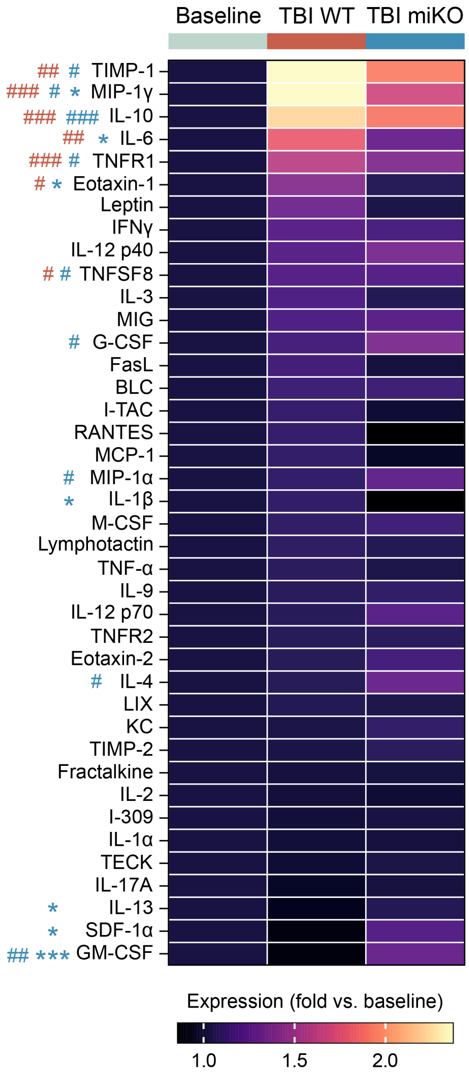


**Figure S3. HDAC3 miKO promotes the resolution of inflammation in the brain after TBI (related to Fig. 3).**

The content of 40 inflammatory cytokines was measured in the brain of HDAC3 miKO mice and WT mice 5 days after TBI. Heatmap shows the mean expression level in each group. The baseline control group was pooled from both WT mice and HDAC3 miKO mice, between which there was no difference (see Figure S1). n=6-7 mice per group. $^{\#}p<0.05$, $^{\#\#}p<0.01$, $^{\#\#\#}p<0.001$ TBI vs. baseline control. $^{*}p<0.05$, $^{***}p<0.001$ HDAC3 miKO vs. WT after TBI.

## Table S1. Key resources

| REAGENT or RESOURCE | SOURCE | IDENTIFIER |
|---|---|---|
| **Antibodies** | | |
| CD45 Monoclonal Antibody (30-F11), eFlour 450 | eBioscience | 48-0451-82 |
| CD11b Monoclonal Antibody (M1/70), APC-eFlour 780 | eBioscience | 47-0112-82 |
| Ly-6G Monoclonal Antibody (1A8-Ly6g), PE | eBioscience | 12-9668-82 |
| CD11c Monoclonal Antibody (N418), PerCP-Cyanine5.5 | eBioscience | 45-0114-82 |
| CD3 Monoclonal Antibody (17A2), APC | eBioscience | 17-0032-82 |
| CD19 Monoclonal Antibody (HIB19), PE-Cyanine7 | eBioscience | 25-0199-42 |
| Rabbit anti-Iba1 | Wako | 019-19741 |
| Rat Anti-Mouse CD16/CD32 Clone 2.4G2 | BD Biosciences | 553142 |
| Mouse MMR/CD206 Antibody | R&D Systems | AF2535 |
| Rabbit Anti-NeuN Antibody | MilliporeSigma | ABN78 |
| Anti-Neurofilament 200 kDa Antibody, clone RT97 | MilliporeSigma | MAB5262 |
| Anti-Neurofilament H (NF-H), Nonphosphorylated Antibody (SMI-32) | BioLegend | 801701 |
| Rabbit beta Amyloid Polyclonal Antibody (CT695) | ThermoFisher | 51-2700 |
| Rat monoclonal Anti-Myelin Basic Protein antibody | Abcam | Ab7349 |
| **Chemicals, Peptides, and Recombinant Proteins** | | |
| Tamoxifen | Sigma-Aldrich | T5648 |
| **Critical Commercial Assays** | | |
| Mouse Inflammation Array C1 | RayBiotech | AAM-INF-1 |
| M.O.M. (Mouse on Mouse) Immunodetection Kit, Basic | Vector Laboratories | BMK-2202 |
| Neural Tissue Dissociation Kit (T) | Miltenyi Biotec | 130-093-231 |
| OneComp eBeads Compensation Beads | ThermoFisher | 01-1111-42 |
| Cell Lysis Buffer | Cell Signaling Technology | 9803 |
| Halt Protease and Phosphatase Inhibitor Cocktail | ThermoFisher | 78842 |
| RNeasy Plus Micro Kit | Qiagen | 74034 |
| RT2 First Strand Kit | Qiagen | 330401 |
| RT2 SYBR Green qPCR Mastermix | Qiagen | 330502 |
| **Experimental Models: Organisms/Strains** | | |
| Mouse: C57BL/6J | The Jackson Laboratory | Stock No: 000664 |
| Mouse: B6;SJL-Tg(ACTFLPe)9205Dym/J | The Jackson Laboratory | Stock No: 003800 |
| Mouse: B6.129P2(Cg)-$Cx3cr1^{tm2.1(cre/ERT2)Litt}$/WganJ | The Jackson Laboratory | Stock No: 021160 |
| Mouse: Hdac3$^{LoxP}$ | This paper | N/A |
| **Oligonucleotides** | | |

| | | |
|---|---|---|
| Genotyping primer: Cx3cr1$^{CreER}$ common GAA CTA CAA TCC TTT AAG GCT CAC G | Ref. [1] | N/A |
| Genotyping primer: Cx3cr1$^{CreER}$ wild type GCA GGA CCT CGG GGT AGT CAC | Ref. [1] | N/A |
| Genotyping primer: Cx3cr1$^{CreER}$ mutant CAC CAG AGA CGG AAA TCC ATC G | Ref. [1] | N/A |
| Genotyping primer: Hdac3$^{LoxP}$ CTG CCG TGG TAT TGG GAA TG | This paper | N/A |
| Genotyping primer: Hdac3$^{LoxP}$ AGA AGG GTG CCA GTG GGT | This paper | N/A |
| qPCR primer: Hdac3 forward CAT CGC CTG GCA TTG ACT CAT | Ref. [2] | N/A |
| qPCR primer: Hdac3 reverse AAG GCA TTA AGG CTC TTG GTG | Ref. [2] | N/A |
| qPCR primer: Gapdh forward GTG AAG GTC GGT GTG AAC GG | Ref. [3] | N/A |
| qPCR primer: Gapdh reverse GTT TCC CGT TGA TGA CCA G | Ref. [3] | N/A |
| **Software and Algorithms** | | |
| FlowJo | FlowJo, LLC | N/A |
| ImageJ | NIH | N/A |
| Imaris 9.6 | Bitplane | N/A |
| Prism 9 | GraphPad | N/A |

**Table S2. The numbers of mice used in this study**

| Figure | | Fig 1 | | Fig 2 | Fig 3 | | Fig 5 | Fig 4-6 |
|---|---|---|---|---|---|---|---|---|
| | **Assay** | **qPCR** | **IHC** | **IHC** | **Antibody array** | **Flow cytometry** | **IHC (β-APP/NF200)** | **Behavior, IHC, TEM and CAP** |
| **0 d** | Baseline WT | 6 | 6 | | | | | |
| | Baseline miKO | 4 | 5 | | | | | |
| **3 d** | Baseline WT | | | 3 | | | | |
| | Baseline miKO | | | 2 | | | | |
| | TBI WT | | | 6 | | | | |
| | TBI miKO | | | 5 | | | | |
| **5 d** | Baseline WT | | | | 3 | | | |
| | Baseline miKO | | | | 4 | | | |
| | TBI WT | | | | 6 | 6 | | |
| | TBI miKO | | | | 6 | 6 | | |
| **7 d** | Baseline WT | | | | | | 4 | |
| | Baseline miKO | | | | | | 4 | |
| | TBI WT | | | | | | 5 | |
| | TBI miKO | | | | | | 6 | |
| **42 d** | Baseline WT | | | | | | | 18 |
| | Baseline miKO | | | | | | | 14 |
| | TBI WT | | | | | | | 31 |
| | TBI miKO | | | | | | | 30 |

## Table S3. Tissue samples processed in this study

| Figure | Assay | Tissue type | Tissue amount per replicate | Number of replicates |
|---|---|---|---|---|
| 1A-1B | Genotyping | Tail biopsy | 2-3 mm of tail | N/A |
| 1C-1E | qPCR | Brain<br>Blood | 7-13×$10^4$ cells sorted from brain or 15-20×$10^4$ cells sorted from blood by FACS | 6 mice (WT)<br>4 mice (miKO) |
| 1F-1G | IHC | Brain | 25-μm thick coronal section | 6 mice (WT)<br>5 mice (miKO) |
| 2 | IHC | Brain | 25-μm thick coronal section | 6 mice (WT)<br>5 mice (miKO) |
| 3A-3B | Antibody array | Brain | Right cerebral hemisphere | 7 mice (baseline)<br>6 mice (TBI WT)<br>6 mice (TBI miKO) |
| 3C-3D | Flow cytometry | Brain | One cerebral hemisphere | 6 mice (WT)<br>6 mice (miKO) |
| 4 | Behavior test | N/A | N/A | See Table S4 |
| 5 | IHC | Brain | 25-μm thick coronal section | See Table S4 |
| 6A-6D | TEM | Brain | 50-nm section cut from 1-$mm^3$ tissue blocks harvested from the corpus callosum | 4 mice (baseline)<br>4 mice (TBI WT)<br>3 mice (TBI miKO) |
| 6E-6G | Electrophysiology | Brain | 350-μm thick coronal slice | 8 mice (baseline WT)<br>5 mice (baseline miKO)<br>8 mice (TBI WT)<br>8 mice (TBI miKO) |

## Table S4. Statistics reporting

| FIGURE | SAMPLE SIZE (n) | DATA STRUCTURE | TEST USED | STATISTIC | P VALUE |
|---|---|---|---|---|---|
| 1E (microglia) | 6 mice (WT)<br>4 mice (miKO) | Normal distribution | *t* test | $t_{(8)} = 14.36$ | miKO-WT < 0.0001 |
| 1E (other brain cells) | 6 mice (WT)<br>4 mice (miKO) | Non-normal distribution | Mann-Whitney test | $U = 6$ | miKO-WT = 0.2571 |
| 1E (monocytes) | 6 mice (WT)<br>4 mice (miKO) | Normal distribution | *t* test | $t_{(8)} = 2.170$ | miKO-WT = 0.0618 |
| 1G (cell number) | 6 mice (WT)<br>5 mice (miKO) | Non-normal distribution | Mann-Whitney test | $U = 0$ | miKO-WT = 0.0043 |
| 1G (cell volume) | 6 mice (WT)<br>5 mice (miKO) | Non-normal distribution | Mann-Whitney test | $U = 4$ | miKO-WT = 0.0519 |
| 1G (surface area) | 6 mice (WT)<br>5 mice (miKO) | Normal distribution | *t* test | $t_{(9)} = 0.9470$ | miKO-WT = 0.3684 |
| 1G (sphericity) | 6 mice (WT)<br>5 mice (miKO) | Normal distribution | *t* test | $t_{(9)} = 2.448$ | miKO-WT = 0.0368 |
| 1G (length A) | 6 mice (WT)<br>5 mice (miKO) | Normal distribution | *t* test | $t_{(9)} = 0.002593$ | miKO-WT = 0.9980 |
| 1G (length C) | 6 mice (WT)<br>5 mice (miKO) | Normal distribution | *t* test | $t_{(9)} = 0.1690$ | miKO-WT = 0.8695 |
| 2E (cortex) | 6 mice (WT)<br>5 mice (miKO) | Normal distribution | Two-way ANOVA; Bonferroni *post hoc* | $F_{(1,18)} = 0.1401$ | ANOVA p=0.7125<br>miKO-WT>0.9999 (0-400 µm)<br>miKO-WT=0.8200 (400-800 µm) |
| 2E (CC) | 6 mice (WT)<br>5 mice (miKO) | Normal distribution | Two-way ANOVA; Bonferroni *post hoc* | $F_{(1,18)} = 5.335$ | ANOVA p=0.0330<br>miKO-WT=0.1183 (0-400 µm)<br>miKO-WT=0.4532 (400-800 µm) |
| 2E (striatum) | 6 mice (WT)<br>5 mice (miKO) | Normal distribution | Two-way ANOVA; Bonferroni *post hoc* | $F_{(1,18)} = 0.4746$ | ANOVA p=0.4997<br>miKO-WT>0.9999 (0-400 µm)<br>miKO-WT=0.5287 (400-800 µm) |
| 2F (cortex, 0-400 µm) | 6 mice (WT)<br>5 mice (miKO) | Normal distribution | *t* test (resting)<br>Welch's *t* test (pro)<br>*t* test (int)<br>*t* test (resolving) | $t_{(9)} = 1.912$ (resting)<br>$t_{(5.722)} = 3.505$ (pro)<br>$t_{(9)} = 0.9953$ (int)<br>$t_{(9)} = 2.350$ (resolving) | miKO-WT = 0.0882 (resting)<br>miKO-WT = 0.0138 (pro)<br>miKO-WT = 0.3456 (int)<br>miKO-WT = 0.0433 (resolving) |
| 2F (cortex, 400-800 µm) | 6 mice (WT)<br>5 mice (miKO) | Normal distribution | *t* test (resting)<br>Welch's *t* test (pro)<br>*t* test (int)<br>Welch's *t* test (resolving) | $t_{(9)} = 3.167$ (resting)<br>$t_{(5.637)} = 2.679$ (pro)<br>$t_{(9)} = 1.384$ (int)<br>$t_{(4.665)} = 1.925$ (resolving) | miKO-WT = 0.0114 (resting)<br>miKO-WT = 0.0389 (pro)<br>miKO-WT = 0.1998 (int)<br>miKO-WT = 0.1164 (resolving) |
| 2F (CC, 0-400 µm) | 6 mice (WT)<br>5 mice (miKO) | Normal distribution (resting, int, resolving)<br>Non-normal distribution (pro) | *t* test (resting)<br>Mann-Whitney test (pro)<br>*t* test (int)<br>*t* test (resolving) | $t_{(9)} = 2.957$ (resting)<br>$U = 9$ (pro)<br>$t_{(9)} = 4.446$ (int)<br>$t_{(9)} = 0.2298$ (resolving) | miKO-WT = 0.0160 (resting)<br>miKO-WT = 0.3290 (pro)<br>miKO-WT = 0.0016 (int)<br>miKO-WT = 0.8234 (resolving) |
| 2F (CC, 400-800 µm) | 6 mice (WT)<br>5 mice (miKO) | Normal distribution (resting, pro, resolving) | *t* test (resting)<br>*t* test (pro)<br>Mann-Whitney test (int)<br>*t* test (resolving) | $t_{(9)} = 0.3967$ (resting)<br>$t_{(9)} = 1.694$ (pro)<br>$U = 12$ (int)<br>$t_{(9)} = 0.8914$ (resolving) | miKO-WT = 0.7009 (resting)<br>miKO-WT = 0.1245 (pro)<br>miKO-WT = 0.6623 (int)<br>miKO-WT = 0.3959 (resolving) |

| | | | | | |
|---|---|---|---|---|---|
| | | Non-normal distribution (int) | | | |
| 2F (striatum, 0-400 µm) | 6 mice (WT)<br>5 mice (miKO) | Normal distribution (resting, int, resolving)<br>Non-normal distribution (pro) | *t* test (resting)<br>Mann-Whitney test (pro)<br>*t* test (int)<br>*t* test (resolving) | $t_{(9)} = 1.039$ (resting)<br>$U = 0$ (pro)<br>$t_{(9)} = 0.5410$ (int)<br>$t_{(9)} = 3.105$ (resolving) | miKO-WT = 0.3260 (resting)<br>miKO-WT = 0.0043 (pro)<br>miKO-WT = 0.6016 (int)<br>miKO-WT = 0.0126 (resolving) |
| 2F (striatum, 400-800 µm) | 6 mice (WT)<br>5 mice (miKO) | Normal distribution | *t* test (resting)<br>*t* test (pro)<br>*t* test (int)<br>*t* test (resolving) | $t_{(9)} = 3.519$ (resting)<br>$t_{(9)} = 1.744$ (pro)<br>$t_{(9)} = 1.008$ (int)<br>$t_{(9)} = 1.618$ (resolving) | miKO-WT = 0.0065 (resting)<br>miKO-WT = 0.1151 (pro)<br>miKO-WT = 0.3397 (int)<br>miKO-WT = 0.1400 (resolving) |
| 3B | See Figure S3 statistics | | | | |
| 3D (neutrophil) | 6 mice (WT)<br>6 mice (miKO) | Non-normal distribution | Mann-Whitney test | $U = 14$ | miKO- WT = 0.5887 |
| 3D (dendritic cell) | 6 mice (WT)<br>6 mice (miKO) | Non-normal distribution | Mann-Whitney test | $U = 12$ | miKO- WT = 0.3939 |
| 3D (microglia) | 6 mice (WT)<br>6 mice (miKO) | Normal distribution | *t* test | $t_{(10)} = 6.674$ | miKO-WT < 0.0001 |
| 3D (macrophage) | 6 mice (WT)<br>6 mice (miKO) | Normal distribution | *t* test | $t_{(10)} = 0.8027$ | miKO-WT = 0.4408 |
| 3D (T cell) | 6 mice (WT)<br>6 mice (miKO) | Normal distribution | *t* test | $t_{(10)} = 1.839$ | miKO-WT = 0.0958 |
| 3D (B cell) | 6 mice (WT)<br>6 mice (miKO) | Normal distribution | *t* test | $t_{(10)} = 1.514$ | miKO-WT = 0.1610 |
| 4A | 9 mice (Baseline WT)<br>9 mice (Baseline miKO)<br>13 mice (TBI WT)<br>13 mice (TBI miKO) | Normal distribution | Two-way RM ANOVA; Bonferroni *post hoc* | $F_{(3, 40)} = 167.4$ | ANOVA p < 0.0001<br>Baseline miKO-Baseline WT > 0.9999<br>TBI WT-Baseline WT < 0.0001<br>TBI miKO-Baseline miKO < 0.0001<br>TBI miKO-TBI WT = 0.1292<br><br>Pre<br>Baseline miKO-Baseline WT > 0.9999<br>TBI WT-Baseline WT > 0.9999<br>TBI miKO-Baseline miKO > 0.9999<br>TBI miKO-TBI WT > 0.9999<br><br>1 day<br>Baseline miKO-Baseline WT > 0.9999<br>TBI WT-Baseline WT < 0.0001<br>TBI miKO-Baseline miKO < 0.0001<br>TBI miKO-TBI WT > 0.9999<br><br>2 days<br>Baseline miKO-Baseline WT > 0.9999<br>TBI WT-Baseline WT < 0.0001<br>TBI miKO-Baseline miKO < 0.0001<br>TBI miKO-TBI WT > 0.9999<br><br>3 days<br>Baseline miKO-Baseline WT > 0.9999<br>TBI WT-Baseline WT < 0.0001<br>TBI miKO-Baseline miKO < 0.0001<br>TBI miKO-TBI WT > 0.9999<br><br>5 days<br>Baseline miKO-Baseline WT > 0.9999<br>TBI WT-Baseline WT < 0.0001 |

| | | | | | |
|---|---|---|---|---|---|
| | | | | | TBI miKO-Baseline miKO < 0.0001<br>TBI miKO-TBI WT = 0.0338<br><br>7 days<br>Baseline miKO-Baseline WT > 0.9999<br>TBI WT-Baseline WT < 0.0001<br>TBI miKO-Baseline miKO < 0.0001<br>TBI miKO-TBI WT = 0.0338<br><br>14 days<br>Baseline miKO-Baseline WT > 0.9999<br>TBI WT-Baseline WT < 0.0001<br>TBI miKO-Baseline miKO < 0.0001<br>TBI miKO-TBI WT = 0.0204<br><br>21 days<br>Baseline miKO-Baseline WT > 0.9999<br>TBI WT-Baseline WT < 0.0001<br>TBI miKO-Baseline miKO = 0.0032<br>TBI miKO-TBI WT = 0.4346<br><br>28 days<br>Baseline miKO-Baseline WT > 0.9999<br>TBI WT-Baseline WT < 0.0001<br>TBI miKO-Baseline miKO = 0.0086<br>TBI miKO-TBI WT = 0.2034<br><br>35 days<br>Baseline miKO-Baseline WT > 0.9999<br>TBI WT-Baseline WT = 0.0089<br>TBI miKO-Baseline miKO = 0.3892<br>TBI miKO-TBI WT = 0.3008 |
| 4B (touch) | 9 mice (Baseline WT)<br>9 mice (Baseline miKO)<br>13 mice (TBI WT)<br>13 mice (TBI miKO) | Normal distribution | Two-way RM ANOVA; Bonferroni *post hoc* | $F_{(3, 40)} = 17.10$ | ANOVA p < 0.0001<br>Baseline miKO-Baseline WT > 0.9999<br>TBI WT-Baseline WT < 0.0001<br>TBI miKO-Baseline miKO = 0.0011<br>TBI miKO-TBI WT = 0.3624<br><br>Pre<br>Baseline miKO-Baseline WT > 0.9999<br>TBI WT-Baseline WT > 0.9999<br>TBI miKO-Baseline miKO > 0.9999<br>TBI miKO-TBI WT > 0.9999<br><br>3 days<br>Baseline miKO-Baseline WT > 0.9999<br>TBI WT-Baseline WT < 0.0001<br>TBI miKO-Baseline miKO < 0.0001<br>TBI miKO-TBI WT < 0.0001<br><br>5 days<br>Baseline miKO-Baseline WT > 0.9999<br>TBI WT-Baseline WT < 0.0001<br>TBI miKO-Baseline miKO < 0.0001<br>TBI miKO-TBI WT > 0.9999<br><br>7 days<br>Baseline miKO-Baseline WT > 0.9999<br>TBI WT-Baseline WT = 0.0003<br>TBI miKO-Baseline miKO = 0.0279<br>TBI miKO-TBI WT = 0.7382 |

| | | | | | |
|---|---|---|---|---|---|
| | | | | | 14 days<br>Baseline miKO-Baseline WT > 0.9999<br>TBI WT-Baseline WT = 0.1012<br>TBI miKO-Baseline miKO = 0.1729<br>TBI miKO-TBI WT > 0.9999<br><br>21 days<br>Baseline miKO-Baseline WT > 0.9999<br>TBI WT-Baseline WT = 0.0795<br>TBI miKO-Baseline miKO = 0.4160<br>TBI miKO-TBI WT > 0.9999<br><br>28 days<br>Baseline miKO-Baseline WT > 0.9999<br>TBI WT-Baseline WT = 0.7544<br>TBI miKO-Baseline miKO = 0.5184<br>TBI miKO-TBI WT > 0.9999<br><br>35 days<br>Baseline miKO-Baseline WT > 0.9999<br>TBI WT-Baseline WT = 0.5424<br>TBI miKO-Baseline miKO > 0.9999<br>TBI miKO-TBI WT > 0.9999 |
| 4B (remove) | 9 mice (Baseline WT)<br>9 mice (Baseline miKO)<br>13 mice (TBI WT)<br>13 mice (TBI miKO) | Normal distribution | Two-way RM ANOVA; Bonferroni *post hoc* | $F_{(3, 40)} = 14.98$ | ANOVA p < 0.0001<br>Baseline miKO-Baseline WT > 0.9999<br>TBI WT-Baseline WT < 0.0001<br>TBI miKO-Baseline miKO = 0.0030<br>TBI miKO-TBI WT = 0.3996<br><br>Pre<br>Baseline miKO-Baseline WT > 0.9999<br>TBI WT-Baseline WT > 0.9999<br>TBI miKO-Baseline miKO > 0.9999<br>TBI miKO-TBI WT > 0.9999<br><br>3 days<br>Baseline miKO-Baseline WT > 0.9999<br>TBI WT-Baseline WT < 0.0001<br>TBI miKO-Baseline miKO < 0.0001<br>TBI miKO-TBI WT < 0.0001<br><br>5 days<br>Baseline miKO-Baseline WT > 0.9999<br>TBI WT-Baseline WT < 0.0001<br>TBI miKO-Baseline miKO < 0.0001<br>TBI miKO-TBI WT > 0.9999<br><br>7 days<br>Baseline miKO-Baseline WT > 0.9999<br>TBI WT-Baseline WT = 0.0002<br>TBI miKO-Baseline miKO = 0.0174<br>TBI miKO-TBI WT = 0.7040<br><br>14 days<br>Baseline miKO-Baseline WT > 0.9999<br>TBI WT-Baseline WT = 0.1240<br>TBI miKO-Baseline miKO = 0.2424<br>TBI miKO-TBI WT > 0.9999<br><br>21 days<br>Baseline miKO-Baseline WT > 0.9999<br>TBI WT-Baseline WT = 0.0316 |

| | | | | | |
|---|---|---|---|---|---|
| | | | | | TBI miKO-Baseline miKO = 0.5485<br>TBI miKO-TBI WT = 0.7488<br><br>28 days<br>Baseline miKO-Baseline WT > 0.9999<br>TBI WT-Baseline WT = 0.6076<br>TBI miKO-Baseline miKO = 0.6157<br>TBI miKO-TBI WT > 0.9999<br><br>35 days<br>Baseline miKO-Baseline WT > 0.9999<br>TBI WT-Baseline WT = 0.4935<br>TBI miKO-Baseline miKO > 0.9999<br>TBI miKO-TBI WT > 0.9999 |
| 4C | 9 mice (Baseline WT)<br>9 mice (Baseline miKO)<br>13 mice (TBI WT)<br>13 mice (TBI miKO) | Normal distribution | Two-way RM ANOVA; Bonferroni *post hoc* | $F_{(3, 40)} = 109.6$ | ANOVA p < 0.0001<br>Baseline miKO-Baseline WT > 0.9999<br>TBI WT-Baseline WT < 0.0001<br>TBI miKO-Baseline miKO < 0.0001<br>TBI miKO-TBI WT = 0.0014<br><br>Pre<br>Baseline miKO-Baseline WT > 0.9999<br>TBI WT-Baseline WT > 0.9999<br>TBI miKO-Baseline miKO > 0.9999<br>TBI miKO-TBI WT > 0.9999<br><br>3 days<br>Baseline miKO-Baseline WT > 0.9999<br>TBI WT-Baseline WT < 0.0001<br>TBI miKO-Baseline miKO < 0.0001<br>TBI miKO-TBI WT = 0.5081<br><br>5 days<br>Baseline miKO-Baseline WT > 0.9999<br>TBI WT-Baseline WT<br>< 0.0001<br>TBI miKO-Baseline miKO < 0.0001<br>TBI miKO-TBI WT > 0.9999<br><br>7 days<br>Baseline miKO-Baseline WT > 0.9999<br>TBI WT-Baseline WT < 0.0001<br>TBI miKO-Baseline miKO < 0.0001<br>TBI miKO-TBI WT = 0.0144<br><br>14 days<br>Baseline miKO-Baseline WT > 0.9999<br>TBI WT-Baseline WT < 0.0001<br>TBI miKO-Baseline miKO < 0.0001<br>TBI miKO-TBI WT = 0.0023<br><br>21 days<br>Baseline miKO-Baseline WT > 0.9999<br>TBI WT-Baseline WT < 0.0001<br>TBI miKO-Baseline miKO < 0.0001<br>TBI miKO-TBI WT = 0.0331<br><br>28 days<br>Baseline miKO-Baseline WT > 0.9999<br>TBI WT-Baseline WT < 0.0001<br>TBI miKO-Baseline miKO < 0.0001<br>TBI miKO-TBI WT < 0.0001 |

| | | | | | |
|---|---|---|---|---|---|
| | | | | | 35 days<br>Baseline miKO-Baseline WT > 0.9999<br>TBI WT-Baseline WT < 0.0001<br>TBI miKO-Baseline miKO < 0.0001<br>TBI miKO-TBI WT = 0.0037 |
| 4D (forepaw) | 9 mice (Baseline WT)<br>9 mice (Baseline miKO)<br>13 mice (TBI WT)<br>13 mice (TBI miKO) | Normal distribution | Mixed-effects model (REML) | $F_{(3, 40)} = 42.43$ | ANOVA p < 0.0001<br>Baseline miKO-Baseline WT > 0.9999<br>TBI WT-Baseline WT < 0.0001<br>TBI miKO-Baseline miKO = 0.0028<br>TBI miKO-TBI WT < 0.0001<br><br>Pre<br>Baseline miKO-Baseline WT > 0.9999<br>TBI WT-Baseline WT > 0.9999<br>TBI miKO-Baseline miKO > 0.9999<br>TBI miKO-TBI WT > 0.9999<br><br>3 days<br>Baseline miKO-Baseline WT > 0.9999<br>TBI WT-Baseline WT < 0.0001<br>TBI miKO-Baseline miKO = 0.0003<br>TBI miKO-TBI WT < 0.0001<br><br>5 days<br>Baseline miKO-Baseline WT > 0.9999<br>TBI WT-Baseline WT < 0.0001<br>TBI miKO-Baseline miKO = 0.0007<br>TBI miKO-TBI WT < 0.0001<br><br>7 days<br>Baseline miKO-Baseline WT > 0.9999<br>TBI WT-Baseline WT < 0.0001<br>TBI miKO-Baseline miKO = 0.0085<br>TBI miKO-TBI WT < 0.0001<br><br>14 days<br>Baseline miKO-Baseline WT > 0.9999<br>TBI WT-Baseline WT < 0.0001<br>TBI miKO-Baseline miKO = 0.0003<br>TBI miKO-TBI WT = 0.0002<br><br>21 days<br>Baseline miKO-Baseline WT > 0.9999<br>TBI WT-Baseline WT < 0.0001<br>TBI miKO-Baseline miKO = 0.0919<br>TBI miKO-TBI WT < 0.0001<br><br>28 days<br>Baseline miKO-Baseline WT > 0.9999<br>TBI WT-Baseline WT < 0.0001<br>TBI miKO-Baseline miKO = 0.2445<br>TBI miKO-TBI WT = 0.0002<br><br>35 days<br>Baseline miKO-Baseline WT > 0.9999<br>TBI WT-Baseline WT < 0.0001<br>TBI miKO-Baseline miKO = 0.1098<br>TBI miKO-TBI WT = 0.0211 |
| 4D (hindpaw) | 9 mice (Baseline WT) | Normal distribution | Mixed-effects model (REML) | $F_{(3, 40)} = 42.87$ | ANOVA p < 0.0001<br>Baseline miKO-Baseline WT > 0.9999<br>TBI WT-Baseline WT < 0.0001 |

| | | | | | |
|---|---|---|---|---|---|
| | 9 mice (Baseline miKO)<br>13 mice (TBI WT)<br>13 mice (TBI miKO) | | | | TBI miKO-Baseline miKO = 0.0003<br>TBI miKO-TBI WT < 0.0001<br><br>Pre<br>Baseline miKO-Baseline WT > 0.9999<br>TBI WT-Baseline WT > 0.9999<br>TBI miKO-Baseline miKO > 0.9999<br>TBI miKO-TBI WT > 0.9999<br><br>3 days<br>Baseline miKO-Baseline WT > 0.9999<br>TBI WT-Baseline WT < 0.0001<br>TBI miKO-Baseline miKO = 0.0057<br>TBI miKO-TBI WT = 0.0005<br><br>5 days<br>Baseline miKO-Baseline WT > 0.9999<br>TBI WT-Baseline WT < 0.0001<br>TBI miKO-Baseline miKO = 0.0155<br>TBI miKO-TBI WT < 0.0001<br><br>7 days<br>Baseline miKO-Baseline WT > 0.9999<br>TBI WT-Baseline WT < 0.0001<br>TBI miKO-Baseline miKO = 0.0054<br>TBI miKO-TBI WT = 0.0477<br><br>14 days<br>Baseline miKO-Baseline WT > 0.9999<br>TBI WT-Baseline WT < 0.0001<br>TBI miKO-Baseline miKO = 0.0015<br>TBI miKO-TBI WT = 0.0089<br><br>21 days<br>Baseline miKO-Baseline WT > 0.9999<br>TBI WT-Baseline WT < 0.0001<br>TBI miKO-Baseline miKO = 0.6530<br>TBI miKO-TBI WT < 0.0001<br><br>28 days<br>Baseline miKO-Baseline WT > 0.9999<br>TBI WT-Baseline WT < 0.0001<br>TBI miKO-Baseline miKO = 0.0039<br>TBI miKO-TBI WT = 0.1273<br><br>35 days<br>Baseline miKO-Baseline WT > 0.9999<br>TBI WT-Baseline WT = 0.0003<br>TBI miKO-Baseline miKO = 0.0551<br>TBI miKO-TBI WT = 0.6653 |
| 4E (learning) | 7 mice (Baseline WT)<br>6 mice (Baseline miKO)<br>13 mice (TBI WT)<br>13 mice (TBI miKO) | Normal distribution | Two-way RM ANOVA; Bonferroni *post hoc* | $F_{(3, 35)} = 7.514$ | ANOVA p = 0.0005<br>Baseline miKO-Baseline WT > 0.9999<br>TBI WT-Baseline WT = 0.0720<br>TBI miKO-Baseline miKO = 0.0432<br>TBI miKO-TBI WT = 0.1880<br><br>29 days<br>Baseline miKO-Baseline WT = 0.0719<br>TBI WT-Baseline WT > 0.9999<br>TBI miKO-Baseline miKO > 0.9999<br>TBI miKO-TBI WT = 0.5646 |

| | | | | | |
|---|---|---|---|---|---|
| | | | | | 30 days<br>Baseline miKO-Baseline WT = 0.8350<br>TBI WT-Baseline WT = 0.0036<br>TBI miKO-Baseline miKO = 0.3235<br>TBI miKO-TBI WT > 0.9999<br><br>31 days<br>Baseline miKO-Baseline WT > 0.9999<br>TBI WT-Baseline WT > 0.9999<br>TBI miKO-Baseline miKO = 0.0610<br>TBI miKO-TBI WT = 0.2238<br><br>32 days<br>Baseline miKO-Baseline WT > 0.9999<br>TBI WT-Baseline WT > 0.9999<br>TBI miKO-Baseline miKO > 0.9999<br>TBI miKO-TBI WT > 0.9999<br><br>33 days<br>Baseline miKO-Baseline WT > 0.9999<br>TBI WT-Baseline WT > 0.9999<br>TBI miKO-Baseline miKO = 0.4299<br>TBI miKO-TBI WT > 0.9999 |
| 4E (target quadrant time) | 7 mice (Baseline WT)<br>6 mice (Baseline miKO)<br>13 mice (TBI WT)<br>13 mice (TBI miKO) | Normal distribution | One-way ANOVA; Bonferroni *post hoc* | $F_{(3, 35)} = 6.370$ | ANOVA p = 0.0015<br>Baseline miKO-Baseline WT > 0.9999<br>TBI WT-Baseline WT = 0.0349<br>TBI miKO-Baseline miKO = 0.1083<br>TBI miKO-TBI WT = 0.3615 |
| 4E (platform crossing) | 7 mice (Baseline WT)<br>6 mice (Baseline miKO)<br>13 mice (TBI WT)<br>13 mice (TBI miKO) | Normal distribution | One-way ANOVA; Bonferroni *post hoc* | $F_{(3, 35)} = 3.912$ | ANOVA p = 0.0165<br>Baseline miKO-Baseline WT > 0.9999<br>TBI WT-Baseline WT = 0.0401<br>TBI miKO-Baseline miKO = 0.6230<br>TBI miKO-TBI WT > 0.9999 |
| 4E (speed) | 7 mice (Baseline WT)<br>6 mice (Baseline miKO)<br>13 mice (TBI WT)<br>13 mice (TBI miKO) | Normal distribution | One-way ANOVA; Bonferroni *post hoc* | $F_{(3, 35)} = 0.2992$ | ANOVA p = 0.8257<br>Baseline miKO-Baseline WT > 0.9999<br>TBI WT-Baseline WT > 0.9999<br>TBI miKO-Baseline miKO > 0.9999<br>TBI miKO-TBI WT > 0.9999 |
| 5C | 5 mice (TBI WT)<br>6 mice (TBI miKO) | Normal distribution | Two-way ANOVA; Bonferroni *post hoc* | $F_{(1, 18)} = 10.42$ | ANOVA p = 0.0047<br>TBI miKO - TBI WT = 0.0004 (CC)<br>TBI miKO - TBI WT > 0.9999 (STR) |
| 5E (7 days cortex) | 4 mice (Baseline WT)<br>4 mice (Baseline miKO)<br>5 mice (TBI WT)<br>6 mice (TBI miKO) | Normal distribution | One-way ANOVA; Bonferroni *post hoc* | $F_{(3, 15)} = 3.475$ | ANOVA p = 0.0428<br>Baseline miKO-Baseline WT > 0.9999<br>TBI WT-Baseline WT = 0.0938<br>TBI miKO-Baseline miKO = 0.8406<br>TBI miKO-TBI WT > 0.9999 |
| 5E (7 days CC) | 4 mice (Baseline WT) | Non-normal distribution | One-way ANOVA on | $H_{(3)} = 5.623$ | ANOVA p = 0.1315<br>Baseline miKO-Baseline WT > 0.9999<br>TBI WT-Baseline WT = 0.4688 |

| | | | | | |
|---|---|---|---|---|---|
| | 4 mice (Baseline miKO)<br>5 mice (TBI WT)<br>6 mice (TBI miKO) | | ranks (Kruskal-Wallis test); Dunn's *post hoc* | | TBI miKO-Baseline miKO > 0.9999<br>TBI miKO-TBI WT > 0.9999 |
| 5E (7 days striatum) | 4 mice (Baseline WT)<br>4 mice (Baseline miKO)<br>5 mice (TBI WT)<br>6 mice (TBI miKO) | Normal distribution | One-way ANOVA; Bonferroni *post hoc* | $F_{(3, 15)} = 14.11$ | ANOVA p = 0.0001<br>Baseline miKO-Baseline WT > 0.9999<br>TBI WT-Baseline WT = 0.0009<br>TBI miKO-Baseline miKO = 0.0050<br>TBI miKO-TBI WT > 0.9999 |
| 5E (42 days cortex) | 4 mice (Baseline WT)<br>4 mice (Baseline miKO)<br>5 mice (TBI WT)<br>5 mice (TBI miKO) | Normal distribution | One-way ANOVA; Bonferroni *post hoc* | $F_{(3, 14)} = 7.814$ | ANOVA p = 0.0026<br>Baseline miKO-Baseline WT > 0.9999<br>TBI WT-Baseline WT = 0.0160<br>TBI miKO-Baseline miKO = 0.0514<br>TBI miKO-TBI WT > 0.9999 |
| 5E (42 days CC) | 4 mice (Baseline WT)<br>4 mice (Baseline miKO)<br>5 mice (TBI WT)<br>5 mice (TBI miKO) | Normal distribution | One-way ANOVA; Bonferroni *post hoc* | $F_{(3, 14)} = 34.35$ | ANOVA p < 0.0001<br>Baseline miKO-Baseline WT > 0.9999<br>TBI WT-Baseline WT < 0.0001<br>TBI miKO-Baseline miKO = 0.0001<br>TBI miKO-TBI WT = 0.3410 |
| 5E (42 days striatum) | 4 mice (Baseline WT)<br>4 mice (Baseline miKO)<br>5 mice (TBI WT)<br>5 mice (TBI miKO) | Normal distribution | One-way ANOVA; Bonferroni *post hoc* | $F_{(3, 14)} = 12.67$ | ANOVA p = 0.0003<br>Baseline miKO-Baseline WT > 0.9999<br>TBI WT-Baseline WT = 0.0010<br>TBI miKO-Baseline miKO = 0.0564<br>TBI miKO-TBI WT = 0.2383 |
| 5G (% axons) | 4 mice (Baseline WT)<br>4 mice (Baseline miKO)<br>5 mice (TBI WT)<br>5 mice (TBI miKO) | Normal distribution | One-way ANOVA; Bonferroni *post hoc* | $F_{(3, 14)} = 18.48$ | ANOVA p < 0.0001<br>Baseline miKO-Baseline WT > 0.9999<br>TBI WT-Baseline WT = 0.0002<br>TBI miKO-Baseline miKO = 0.0147<br>TBI miKO-TBI WT = 0.0707 |
| 5G (% baseline) | 5 mice (TBI WT)<br>5 mice (TBI miKO) | Normal distribution | *t* test | $t_{(8)} = 2.475$ | TBI miKO-TBI WT = 0.0384 |
| 5H | 12 mice (TBI WT)<br>12 mice (TBI miKO) | Normal distribution | *t* test | $t_{(22)} = 2.480$ | TBI miKO-TBI WT = 0.0213 |
| 6B (myelinated) | 4 mice (TBI WT)<br>3 mice (TBI miKO) | Normal distribution | *t* test | $t_{(5)} = 0.08965$ | TBI miKO-TBI WT = 0.9320 |
| 6B (demyelinated) | 4 mice (TBI WT)<br>3 mice (TBI miKO) | Normal distribution | *t* test | $t_{(5)} = 0.3944$ | TBI miKO-TBI WT = 0.7096 |

| | | | | | |
|---|---|---|---|---|---|
| 6C (%) | 4 mice (TBI WT)<br>3 mice (TBI miKO) | Normal distribution | *t* test | $t_{(5)} = 3.589$ | TBI miKO-TBI WT = 0.0157 |
| 6C (number per FOV) | 4 mice (TBI WT)<br>3 mice (TBI miKO) | Normal distribution | Two-way ANOVA; Bonferroni *post hoc* | $F_{(2, 15)} = 19.72$ | ANOVA p < 0.0001<br>TBI miKO - TBI WT > 0.9999 (outfolds)<br>TBI miKO - TBI WT < 0.0001 (splitting)<br>TBI miKO - TBI WT = 0.7510 (vacuolization) |
| 6D | 4 mice (Baseline)<br>4 mice (TBI WT)<br>3 mice (TBI miKO) | Normal distribution | Two-way ANOVA; Bonferroni *post hoc* | $F_{(2, 24)} = 82.45$ | ANOVA p < 0.0001<br><br>0.2-0.4<br>TBI WT - Baseline < 0.0001<br>TBI miKO - Baseline = 0.0001<br>TBI miKO - TBI WT = 0.0651<br><br>0.4-0.6<br>TBI miKO - Baseline < 0.0001<br>TBI miKO - Baseline = 0.0024<br>TBI miKO - TBI WT = 0.0217<br><br>> 0.6<br>TBI miKO - Baseline < 0.0001<br>TBI miKO - Baseline = 0.0645<br>TBI miKO - TBI WT = 0.0021 |
| 6G (0.75 mm N1) | 8 mice (Baseline WT)<br>5 mice (Baseline miKO)<br>8 mice (TBI WT)<br>8 mice (TBI miKO) | Normal distribution | Two-way ANOVA; Bonferroni *post hoc* | $F_{(3, 225)} = 34.91$ | ANOVA p < 0.0001<br>Baseline miKO-Baseline WT > 0.9999<br>TBI WT-Baseline WT < 0.0001<br>TBI miKO-Baseline miKO > 0.9999<br>TBI miKO-TBI WT < 0.0001<br><br>0 µA<br>Baseline miKO-Baseline WT > 0.9999<br>TBI WT-Baseline WT > 0.9999<br>TBI miKO-Baseline miKO > 0.9999<br>TBI miKO-TBI WT > 0.9999<br><br>250 µA<br>Baseline miKO-Baseline WT > 0.9999<br>TBI WT-Baseline WT = 0.0011<br>TBI miKO-Baseline miKO > 0.9999<br>TBI miKO-TBI WT = 0.1807<br><br>500 µA<br>Baseline miKO-Baseline WT > 0.9999<br>TBI WT-Baseline WT = 0.0008<br>TBI miKO-Baseline miKO > 0.9999<br>TBI miKO-TBI WT = 0.0576<br><br>750 µA<br>Baseline miKO-Baseline WT > 0.9999<br>TBI WT-Baseline WT = 0.0014<br>TBI miKO-Baseline miKO > 0.9999<br>TBI miKO-TBI WT = 0.0222<br><br>1000 µA<br>Baseline miKO-Baseline WT > 0.9999<br>TBI WT-Baseline WT = 0.0018<br>TBI miKO-Baseline miKO > 0.9999<br>TBI miKO-TBI WT = 0.0185 |

| | | | | | |
|---|---|---|---|---|---|
| | | | | | 1250 µA<br>Baseline miKO-Baseline WT > 0.9999<br>TBI WT-Baseline WT = 0.0032<br>TBI miKO-Baseline miKO > 0.9999<br>TBI miKO-TBI WT = 0.0605<br><br>1500 µA<br>Baseline miKO-Baseline WT > 0.9999<br>TBI WT-Baseline WT = 0.0052<br>TBI miKO-Baseline miKO > 0.9999<br>TBI miKO-TBI WT = 0.0513<br><br>1750 µA<br>Baseline miKO-Baseline WT > 0.9999<br>TBI WT-Baseline WT = 0.0084<br>TBI miKO-Baseline miKO = 0.8700<br>TBI miKO-TBI WT = 0.0440<br><br>2000 µA<br>Baseline miKO-Baseline WT > 0.9999<br>TBI WT-Baseline WT = 0.0091<br>TBI miKO-Baseline miKO > 0.9999<br>TBI miKO-TBI WT = 0.0243 |
| 6G (0.75 mm N2) | 8 mice (Baseline WT)<br>5 mice (Baseline miKO)<br>8 mice (TBI WT)<br>8 mice (TBI miKO) | Normal distribution | Two-way ANOVA; Bonferroni *post hoc* | $F_{(3, 225)} = 102.1$ | ANOVA p < 0.0001<br>Baseline miKO-Baseline WT > 0.9999<br>TBI WT-Baseline WT < 0.0001<br>TBI miKO-Baseline miKO < 0.0001<br>TBI miKO-TBI WT < 0.0001<br><br>0 µA<br>Baseline miKO-Baseline WT > 0.9999<br>TBI WT-Baseline WT > 0.9999<br>TBI miKO-Baseline miKO > 0.9999<br>TBI miKO-TBI WT > 0.9999<br><br>250 µA<br>Baseline miKO-Baseline WT > 0.9999<br>TBI WT-Baseline WT < 0.0001<br>TBI miKO-Baseline miKO = 0.2090<br>TBI miKO-TBI WT > 0.9999<br><br>500 µA<br>Baseline miKO-Baseline WT > 0.9999<br>TBI WT-Baseline WT < 0.0001<br>TBI miKO-Baseline miKO = 0.0032<br>TBI miKO-TBI WT = 0.4927<br><br>750 µA<br>Baseline miKO-Baseline WT > 0.9999<br>TBI WT-Baseline WT < 0.0001<br>TBI miKO-Baseline miKO = 0.0077<br>TBI miKO-TBI WT = 0.1973<br><br>1000 µA<br>Baseline miKO-Baseline WT > 0.9999<br>TBI WT-Baseline WT < 0.0001<br>TBI miKO-Baseline miKO = 0.0060<br>TBI miKO-TBI WT = 0.1943<br><br>1250 µA<br>Baseline miKO-Baseline WT > 0.9999 |

| | | | | | |
|---|---|---|---|---|---|
| | | | | | TBI WT-Baseline WT < 0.0001<br>TBI miKO-Baseline miKO = 0.0072<br>TBI miKO-TBI WT = 0.2768<br><br>1500 µA<br>Baseline miKO-Baseline WT > 0.9999<br>TBI WT-Baseline WT < 0.0001<br>TBI miKO-Baseline miKO = 0.0144<br>TBI miKO-TBI WT = 0.3282<br><br>1750 µA<br>Baseline miKO-Baseline WT > 0.9999<br>TBI WT-Baseline WT < 0.0001<br>TBI miKO-Baseline miKO = 0.0100<br>TBI miKO-TBI WT = 0.2582<br><br>2000 µA<br>Baseline miKO-Baseline WT > 0.9999<br>TBI WT-Baseline WT < 0.0001<br>TBI miKO-Baseline miKO = 0.0147<br>TBI miKO-TBI WT = 0.1732 |
| 6G (1 mm N1) | 8 mice (Baseline WT)<br>5 mice (Baseline miKO)<br>8 mice (TBI WT)<br>8 mice (TBI miKO) | Normal distribution | Two-way ANOVA; Bonferroni *post hoc* | $F_{(3, 225)} = 46.75$ | ANOVA p < 0.0001<br>Baseline miKO-Baseline WT > 0.9999<br>TBI WT-Baseline WT < 0.0001<br>TBI miKO-Baseline miKO = 0.0023<br>TBI miKO-TBI WT < 0.0001<br><br>0 µA<br>Baseline miKO-Baseline WT > 0.9999<br>TBI WT-Baseline WT > 0.9999<br>TBI miKO-Baseline miKO > 0.9999<br>TBI miKO-TBI WT > 0.9999<br><br>250 µA<br>Baseline miKO-Baseline WT > 0.9999<br>TBI WT-Baseline WT = 0.0003<br>TBI miKO-Baseline miKO > 0.9999<br>TBI miKO-TBI WT = 0.4661<br><br>500 µA<br>Baseline miKO-Baseline WT > 0.9999<br>TBI WT-Baseline WT = 0.0001<br>TBI miKO-Baseline miKO > 0.9999<br>TBI miKO-TBI WT = 0.1302<br><br>750 µA<br>Baseline miKO-Baseline WT > 0.9999<br>TBI WT-Baseline WT = 0.0004<br>TBI miKO-Baseline miKO > 0.9999<br>TBI miKO-TBI WT = 0.1245<br><br>1000 µA<br>Baseline miKO-Baseline WT > 0.9999<br>TBI WT-Baseline WT = 0.0001<br>TBI miKO-Baseline miKO = 0.7784<br>TBI miKO-TBI WT = 0.0792<br><br>1250 µA<br>Baseline miKO-Baseline WT > 0.9999<br>TBI WT-Baseline WT = 0.0005<br>TBI miKO-Baseline miKO = 0.8038<br>TBI miKO-TBI WT = 0.1200 |

| | | | | | |
|---|---|---|---|---|---|
| | | | | | 1500 μA<br>Baseline miKO-Baseline WT > 0.9999<br>TBI WT-Baseline WT = 0.0004<br>TBI miKO-Baseline miKO = 0.6088<br>TBI miKO-TBI WT = 0.1469<br><br>1750 μA<br>Baseline miKO-Baseline WT > 0.9999<br>TBI WT-Baseline WT = 0.0009<br>TBI miKO-Baseline miKO = 0.8700<br>TBI miKO-TBI WT = 0.1344<br><br>2000 μA<br>Baseline miKO-Baseline WT > 0.9999<br>TBI WT-Baseline WT = 0.0011<br>TBI miKO-Baseline miKO > 0.9999<br>TBI miKO-TBI WT = 0.1147 |
| 6G (1mm N2) | 8 mice (Baseline WT)<br>5 mice (Baseline miKO)<br>8 mice (TBI WT)<br>8 mice (TBI miKO) | Normal distribution | Two-way ANOVA; Bonferroni *post hoc* | $F_{(3, 225)} = 107.7$ | ANOVA p < 0.0001<br>Baseline miKO-Baseline WT > 0.9999<br>TBI WT-Baseline WT < 0.0001<br>TBI miKO-Baseline miKO < 0.0001<br>TBI miKO-TBI WT < 0.0001<br><br>0 μA<br>Baseline miKO-Baseline WT > 0.9999<br>TBI WT-Baseline WT > 0.9999<br>TBI miKO-Baseline miKO > 0.9999<br>TBI miKO-TBI WT > 0.9999<br><br>250 μA<br>Baseline miKO-Baseline WT = 0.5633<br>TBI WT-Baseline WT < 0.0001<br>TBI miKO-Baseline miKO = 0.1112<br>TBI miKO-TBI WT > 0.9999<br><br>500 μA<br>Baseline miKO-Baseline WT > 0.9999<br>TBI WT-Baseline WT < 0.0001<br>TBI miKO-Baseline miKO = 0.0716<br>TBI miKO-TBI WT = 0.1388<br><br>750 μA<br>Baseline miKO-Baseline WT > 0.9999<br>TBI WT-Baseline WT < 0.0001<br>TBI miKO-Baseline miKO = 0.0348<br>TBI miKO-TBI WT = 0.0658<br><br>1000 μA<br>Baseline miKO-Baseline WT > 0.9999<br>TBI WT-Baseline WT < 0.0001<br>TBI miKO-Baseline miKO = 0.0402<br>TBI miKO-TBI WT = 0.0321<br><br>1250 μA<br>Baseline miKO-Baseline WT > 0.9999<br>TBI WT-Baseline WT < 0.0001<br>TBI miKO-Baseline miKO = 0.0286<br>TBI miKO-TBI WT = 0.0422<br><br>1500 μA<br>Baseline miKO-Baseline WT > 0.9999 |

| | | | | | |
|---|---|---|---|---|---|
| | | | | | TBI WT-Baseline WT < 0.0001<br>TBI miKO-Baseline miKO = 0.0076<br>TBI miKO-TBI WT = 0.0239<br><br>1750 μA<br>Baseline miKO-Baseline WT > 0.9999<br>TBI WT-Baseline WT < 0.0001<br>TBI miKO-Baseline miKO = 0.0159<br>TBI miKO-TBI WT = 0.0536<br><br>2000 μA<br>Baseline miKO-Baseline WT > 0.9999<br>TBI WT-Baseline WT < 0.0001<br>TBI miKO-Baseline miKO = 0.0146<br>TBI miKO-TBI WT = 0.0821 |
| S1 | 3 mice (WT)<br>4 mice (miKO) | Normal distribution<br><br>Non-normal distribution (G-CSF, IL-2, IL-4, I-309, TNFR2) | *t* test<br><br>Mann-Whitney test (G-CSF, IL-2, IL-4, I-309, TNFR2) | $t_{(5)}$ = 1.327 (BLC)<br>$t_{(5)}$ = 2.446 (TNFSF8)<br>$t_{(5)}$ = 0.6449 (Eotaxin-1)<br>$t_{(5)}$ = 2.094 (Eotaxin-2)<br>$t_{(5)}$ = 1.385 (FasL)<br>$t_{(5)}$ = 2.019 (fractalkine)<br>$U$ = 2 (G-CSF)<br>$t_{(5)}$ = 1.155 (GM-CSF)<br>$t_{(5)}$ = 1.071 (IFNγ)<br>$t_{(5)}$ = 2.035 (IL-1α)<br>$t_{(5)}$ = 1.540 (IL-1β)<br>$U$ = 6 (IL-2)<br>$t_{(5)}$ = 0.1200 (IL-3)<br>$U$ = 3 (IL-4)<br>$t_{(5)}$ = 2.086 (IL-6)<br>$t_{(5)}$ = 1.638 (IL-9)<br>$t_{(5)}$ = 0.07522 (IL-10)<br>$t_{(5)}$ = 0.3819 (IL-12 p40)<br>$t_{(5)}$ = 0.1104 (IL-12 p70)<br>$t_{(5)}$ = 1.177 (IL-13)<br>$t_{(5)}$ = 1.299 (IL-17A)<br>$t_{(5)}$ = 0.1916 (I-TAC)<br>$t_{(5)}$ = 1.961 (KC)<br>$t_{(5)}$ = 0.8649 (Leptin)<br>$t_{(5)}$ = 2.304 (LIX)<br>$t_{(5)}$ = 0.9250 (Lymphotactin)<br>$t_{(5)}$ = 0.04243 (MCP-1)<br>$t_{(5)}$ = 0.5086 (M-CSF)<br>$t_{(5)}$ = 0.2656 (MIG)<br>$t_{(5)}$ = 2.169 (MIP-1α)<br>$t_{(5)}$ = 0.7612 (MIP-1γ) | miKO-WT = 0.2417 (BLC)<br>miKO-WT = 0.0582 (TNFSF8)<br>miKO-WT = 0.5474 (Eotaxin-1)<br>miKO-WT = 0.0904 (Eotaxin-2)<br>miKO-WT = 0.2248 (FasL)<br>miKO-WT = 0.0995 (fractalkine)<br>miKO-WT = 0.2286 (G-CSF)<br>miKO-WT = 0.3003 (GM-CSF)<br>miKO-WT = 0.3329 (IFNγ)<br>miKO-WT = 0.0975 (IL-1α)<br>miKO-WT = 0.1843 (IL-1β)<br>miKO-WT > 0.9999 (IL-2)<br>miKO-WT = 0.9092 (IL-3)<br>miKO-WT = 0.4000 (IL-4)<br>miKO-WT = 0.0913 (IL-6)<br>miKO-WT = 0.1624 (IL-9)<br>miKO-WT = 0.9430 (IL-10)<br>miKO-WT = 0.7182 (IL-12 p40)<br>miKO-WT = 0.9164 (IL-12 p70)<br>miKO-WT = 0.2922 (IL-13)<br>miKO-WT = 0.2508 (IL-17A)<br>miKO-WT = 0.8556 (I-TAC)<br>miKO-WT = 0.1071 (KC)<br>miKO-WT = 0.4266 (Leptin)<br>miKO-WT = 0.0694 (LIX)<br>miKO-WT = 0.3974 (Lymphotactin)<br>miKO-WT = 0.9678 (MCP-1)<br>miKO-WT = 0.6326 (M-CSF)<br>miKO-WT = 0.8011 (MIG)<br>miKO-WT = 0.0822 (MIP-1α)<br>miKO-WT = 0.4809 (MIP-1γ)<br>miKO-WT = 0.9318 (RANTES)<br>miKO-WT = 0.2501 (SDF-1α)<br>miKO-WT = 0.2286 (I-309)<br>miKO-WT = 0.6831 (TECK)<br>miKO-WT = 0.8140 (TIMP-1)<br>miKO-WT = 0.8920 (TIMP-2)<br>miKO-WT = 0.8855 (TNF-α)<br>miKO-WT = 0.4471 (TNFR1)<br>miKO-WT = 0.4000 (TNFR2) |

| | | | | | |
|---|---|---|---|---|---|
| | | | | $t_{(5)}$ = 0.08992 (RANTES)<br>$t_{(5)}$ = 1.301 (SDF-1α)<br>$U$ = 2 (I-309)<br>$t_{(5)}$ = 0.4330 (TECK)<br>$t_{(5)}$ = 0.2480 (TIMP-1)<br>$t_{(5)}$ = 0.1428 (TIMP-2)<br>$t_{(5)}$ = 0.1515 (TNF-α)<br>$t_{(5)}$ = 0.8247 (TNFR1)<br>$U$ = 3 (TNFR2) | |
| S2 (TIMP-1) | 7 mice (Baseline)<br>6 mice (TBI WT)<br>6 mice (TBI miKO) | Normal distribution | One-way ANOVA; Bonferroni *post hoc* | $F_{(2, 16)}$ = 10.09 | ANOVA p = 0.0015<br>TBI WT-Baseline = 0.0014<br>TBI miKO-Baseline = 0.0288<br>TBI miKO-TBI WT = 0.5614 |
| S2 (MIP-1γ) | 7 mice (Baseline)<br>6 mice (TBI WT)<br>6 mice (TBI miKO) | Normal distribution | One-way ANOVA; Bonferroni *post hoc* | $F_{(2, 16)}$ = 15.21 | ANOVA p = 0.0002<br>TBI WT-Baseline = 0.0001<br>TBI miKO-Baseline = 0.0483<br>TBI miKO-TBI WT = 0.0454 |
| S2 (IL-10) | 7 mice (Baseline)<br>6 mice (TBI WT)<br>6 mice (TBI miKO) | Normal distribution | One-way ANOVA; Bonferroni *post hoc* | $F_{(2, 16)}$ = 26.18 | ANOVA p < 0.0001<br>TBI WT-Baseline < 0.0001<br>TBI miKO-Baseline = 0.0004<br>TBI miKO-TBI WT = 0.2582 |
| S2 (IL-6) | 7 mice (Baseline)<br>6 mice (TBI WT)<br>6 mice (TBI miKO) | Normal distribution | One-way ANOVA; Bonferroni *post hoc* | $F_{(2, 16)}$ = 9.400 | ANOVA p = 0.0020<br>TBI WT-Baseline = 0.0017<br>TBI miKO-Baseline = 0.5020<br>TBI miKO-TBI WT = 0.0433 |
| S2 (TNFR1) | 7 mice (Baseline)<br>6 mice (TBI WT)<br>6 mice (TBI miKO) | Normal distribution | One-way ANOVA; Bonferroni *post hoc* | $F_{(2, 16)}$ = 15.02 | ANOVA p = 0.0002<br>TBI WT-Baseline = 0.0002<br>TBI miKO-Baseline = 0.0113<br>TBI miKO-TBI WT = 0.2157 |
| S2 (Eotaxin-1) | 7 mice (Baseline)<br>6 mice (TBI WT)<br>6 mice (TBI miKO) | Normal distribution | One-way ANOVA; Bonferroni *post hoc* | $F_{(2, 16)}$ = 6.052 | ANOVA p = 0.0110<br>TBI WT-Baseline = 0.0153<br>TBI miKO-Baseline > 0.9999<br>TBI miKO-TBI WT = 0.0419 |
| S2 (Leptin) | 7 mice (Baseline)<br>6 mice (TBI WT)<br>6 mice (TBI miKO) | Normal distribution | One-way ANOVA; Bonferroni *post hoc* | $F_{(2, 16)}$ = 2.632 | ANOVA p = 0.1027<br>TBI WT-Baseline = 0.1664<br>TBI miKO-Baseline > 0.9999<br>TBI miKO-TBI WT = 0.2163 |
| S2 (IFNγ) | 7 mice (Baseline)<br>6 mice (TBI WT) | Normal distribution | One-way ANOVA; Bonferroni *post hoc* | $F_{(2, 16)}$ = 1.317 | ANOVA p = 0.2953<br>TBI WT-Baseline = 0.4215<br>TBI miKO-Baseline= 0.8119<br>TBI miKO-TBI WT > 0.9999 |

| | | | | | |
|---|---|---|---|---|---|
| | 6 mice (TBI miKO) | | | | |
| S2 (IL-12 p40) | 7 mice (Baseline)<br>6 mice (TBI WT)<br>6 mice (TBI miKO) | Normal distribution | One-way ANOVA; Bonferroni *post hoc* | $F_{(2, 16)} = 1.890$ | ANOVA p = 0.1833<br>TBI WT-Baseline = 0.7734<br>TBI miKO-Baseline = 0.2199<br>TBI miKO-TBI WT > 0.9999 |
| S2 (TNFSF8) | 7 mice (Baseline)<br>6 mice (TBI WT)<br>6 mice (TBI miKO) | Normal distribution | One-way ANOVA; Bonferroni *post hoc* | $F_{(2, 16)} = 5.610$ | ANOVA p = 0.0143<br>TBI WT-Baseline = 0.0347<br>TBI miKO-Baseline = 0.0329<br>TBI miKO-TBI WT > 0.9999 |
| S2 (IL-3) | 7 mice (Baseline)<br>6 mice (TBI WT)<br>6 mice (TBI miKO) | Normal distribution | One-way ANOVA; Bonferroni *post hoc* | $F_{(2, 16)} = 1.772$ | ANOVA p = 0.2018<br>TBI WT-Baseline = 0.2664<br>TBI miKO-Baseline > 0.9999<br>TBI miKO-TBI WT = 0.5626 |
| S2 (MIG) | 7 mice (Baseline)<br>6 mice (TBI WT)<br>6 mice (TBI miKO) | Normal distribution | One-way ANOVA; Bonferroni *post hoc* | $F_{(2, 16)} = 1.745$ | ANOVA p = 0.2062<br>TBI WT-Baseline = 0.5277<br>TBI miKO-Baseline = 0.3061<br>TBI miKO-TBI WT > 0.9999 |
| S2 (G-CSF) | 7 mice (Baseline)<br>6 mice (TBI WT)<br>6 mice (TBI miKO) | Normal distribution | One-way ANOVA; Bonferroni *post hoc* | $F_{(2, 16)} = 4.680$ | ANOVA p = 0.0251<br>TBI WT-Baseline = 0.6348<br>TBI miKO-Baseline = 0.0226<br>TBI miKO-TBI WT = 0.3302 |
| S2 (Fasl) | 7 mice (Baseline)<br>6 mice (TBI WT)<br>6 mice (TBI miKO) | Normal distribution | One-way ANOVA; Bonferroni *post hoc* | $F_{(2, 16)} = 2.082$ | ANOVA p = 0.1572<br>TBI WT-Baseline = 0.3144<br>TBI miKO-Baseline > 0.9999<br>TBI miKO-TBI WT = 0.2573 |
| S2 (BLC) | 7 mice (Baseline)<br>6 mice (TBI WT)<br>6 mice (TBI miKO) | Normal distribution | One-way ANOVA; Bonferroni *post hoc* | $F_{(2, 16)} = 3.525$ | ANOVA p = 0.0539<br>TBI WT-Baseline = 0.1184<br>TBI miKO-Baseline = 0.1065<br>TBI miKO-TBI WT > 0.9999 |
| S2 (I-TAC) | 7 mice (Baseline)<br>6 mice (TBI WT)<br>6 mice (TBI miKO) | Normal distribution | One-way ANOVA; Bonferroni *post hoc* | $F_{(2, 16)} = 1.817$ | ANOVA p = 0.1945<br>TBI WT-Baseline = 0.5151<br>TBI miKO-Baseline > 0.9999<br>TBI miKO-TBI WT = 0.2629 |
| S2 (RANTES) | 7 mice (Baseline)<br>6 mice (TBI WT)<br>6 mice (TBI miKO) | Normal distribution | One-way ANOVA; Bonferroni *post hoc* | $F_{(2, 16)} = 2.126$ | ANOVA p = 0.1518<br>TBI WT-Baseline > 0.9999<br>TBI miKO-Baseline = 0.6431<br>TBI miKO-TBI WT = 0.1734 |
| S2 (MCP-1) | 7 mice (Baseline)<br>6 mice (TBI WT)<br>6 mice (TBI miKO) | Normal distribution | One-way ANOVA; Bonferroni *post hoc* | $F_{(2, 16)} = 2.435$ | ANOVA p = 0.1193<br>TBI WT-Baseline = 0.6015<br>TBI miKO-Baseline > 0.9999<br>TBI miKO-TBI WT = 0.1298 |

| | | | | | |
|---|---|---|---|---|---|
| S2 (MIP-1α) | 7 mice (Baseline)<br>6 mice (TBI WT)<br>6 mice (TBI miKO) | Normal distribution | One-way ANOVA; Bonferroni *post hoc* | $F_{(2, 16)} = 4.267$ | ANOVA p = 0.0327<br>TBI WT-Baseline = 0.9150<br>TBI miKO-Baseline = 0.0311<br>TBI miKO-TBI WT = 0.2836 |
| S2 (IL-1β) | 7 mice (Baseline)<br>6 mice (TBI WT)<br>6 mice (TBI miKO) | Normal distribution | One-way ANOVA; Bonferroni *post hoc* | $F_{(2, 16)} = 6.018$ | ANOVA p = 0.0113<br>TBI WT-Baseline = 0.6090<br>TBI miKO-Baseline = 0.1211<br>TBI miKO-TBI WT = 0.0103 |
| S2 (M-CSF) | 7 mice (Baseline)<br>6 mice (TBI WT)<br>6 mice (TBI miKO) | Normal distribution | One-way ANOVA; Bonferroni *post hoc* | $F_{(2, 16)} = 2.133$ | ANOVA p = 0.1510<br>TBI WT-Baseline = 0.6137<br>TBI miKO-Baseline = 0.1822<br>TBI miKO-TBI WT > 0.9999 |
| S2 (Lymphotactin) | 7 mice (Baseline)<br>6 mice (TBI WT)<br>6 mice (TBI miKO) | Normal distribution | One-way ANOVA; Bonferroni *post hoc* | $F_{(2, 16)} = 1.367$ | ANOVA p = 0.2832<br>TBI WT-Baseline = 0.3533<br>TBI miKO-Baseline > 0.9999<br>TBI miKO-TBI WT > 0.9999 |
| S2 (TNF-α) | 7 mice (Baseline)<br>6 mice (TBI WT)<br>6 mice (TBI miKO) | Normal distribution | One-way ANOVA; Bonferroni *post hoc* | $F_{(2, 16)} = 0.3230$ | ANOVA p = 0.7286<br>TBI WT-Baseline > 0.9999<br>TBI miKO-Baseline > 0.9999<br>TBI miKO-TBI WT > 0.9999 |
| S2 (IL-9) | 7 mice (Baseline)<br>6 mice (TBI WT)<br>6 mice (TBI miKO) | Normal distribution | One-way ANOVA; Bonferroni *post hoc* | $F_{(2, 16)} = 0.3981$ | ANOVA p = 0.6781<br>TBI WT-Baseline > 0.9999<br>TBI miKO-Baseline > 0.9999<br>TBI miKO-TBI WT > 0.9999 |
| S2 (IL-12 p70) | 7 mice (Baseline)<br>6 mice (TBI WT)<br>6 mice (TBI miKO) | Normal distribution | One-way ANOVA; Bonferroni *post hoc* | $F_{(2, 16)} = 1.613$ | ANOVA p = 0.2301<br>TBI WT-Baseline > 0.9999<br>TBI miKO-Baseline= 0.2990<br>TBI miKO-TBI WT = 0.6772 |
| S2 (TNFR2) | 7 mice (Baseline)<br>6 mice (TBI WT)<br>6 mice (TBI miKO) | Non-normal distribution | One-way ANOVA on ranks (Kruskal-Wallis test); Dunn's *post hoc* | $H_{(2)} = 3.910$ | ANOVA p = 0.1422<br>TBI WT-Baseline = 0.1440<br>TBI miKO-Baseline > 0.9999<br>TBI miKO-TBI WT = 0.9147 |
| S2 (Eotaxin-2) | 7 mice (Baseline)<br>6 mice (TBI WT)<br>6 mice (TBI miKO) | Non-normal distribution | One-way ANOVA on ranks (Kruskal-Wallis test); Dunn's *post hoc* | $H_{(2)} = 2.383$ | ANOVA p = 0.3154<br>TBI WT-Baseline > 0.9999<br>TBI miKO-Baseline = 0.3848<br>TBI miKO-TBI WT > 0.9999 |
| S2 (IL-4) | 7 mice (Baseline)<br>6 mice (TBI WT)<br>6 mice (TBI miKO) | Normal distribution | One-way ANOVA; Bonferroni *post hoc* | $F_{(2, 16)} = 5.884$ | ANOVA p = 0.0121<br>TBI WT-Baseline > 0.9999<br>TBI miKO-Baseline = 0.0146<br>TBI miKO-TBI WT = 0.0586 |
| S2 (LIX) | 7 mice (Baseline) | Non-normal distribution | One-way ANOVA on | $H_{(2)} = 1.231$ | ANOVA p = 0.5565<br>TBI WT-Baseline = 0.9245 |

| | | | | | |
|---|---|---|---|---|---|
| | 6 mice (TBI WT)<br>6 mice (TBI miKO) | | ranks (Kruskal-Wallis test); Dunn's *post hoc* | | TBI miKO-Baseline > 0.9999<br>TBI miKO-TBI WT > 0.9999 |
| S2 (KC) | 7 mice (Baseline)<br>6 mice (TBI WT)<br>6 mice (TBI miKO) | Normal distribution | One-way ANOVA; Bonferroni *post hoc* | $F_{(2, 16)} = 1.968$ | ANOVA p = 0.1721<br>TBI WT-Baseline > 0.9999<br>TBI miKO-Baseline= 0.2434<br>TBI miKO-TBI WT = 0.4201 |
| S2 (TIMP-2) | 7 mice (Baseline)<br>6 mice (TBI WT)<br>6 mice (TBI miKO) | Normal distribution | One-way ANOVA; Bonferroni *post hoc* | $F_{(2, 16)} = 0.4612$ | ANOVA p = 0.6386<br>TBI WT-Baseline > 0.9999<br>TBI miKO-Baseline > 0.9999<br>TBI miKO-TBI WT > 0.9999 |
| S2 (Fractalkine) | 7 mice (Baseline)<br>6 mice (TBI WT)<br>6 mice (TBI miKO) | Normal distribution | One-way ANOVA; Bonferroni *post hoc* | $F_{(2, 16)} = 0.04304$ | ANOVA p = 0.9580<br>TBI WT-Baseline > 0.9999<br>TBI miKO-Baseline > 0.9999<br>TBI miKO-TBI WT > 0.9999 |
| S2 (IL-2) | 7 mice (Baseline)<br>6 mice (TBI WT)<br>6 mice (TBI miKO) | Normal distribution | One-way ANOVA; Bonferroni *post hoc* | $F_{(2, 16)} = 0.1173$ | ANOVA p = 0.8900<br>TBI WT-Baseline > 0.9999<br>TBI miKO-Baseline > 0.9999<br>TBI miKO-TBI WT > 0.9999 |
| S2 (I-309) | 7 mice (Baseline)<br>6 mice (TBI WT)<br>6 mice (TBI miKO) | Non-normal distribution | One-way ANOVA on ranks (Kruskal-Wallis test); Dunn's *post hoc* | $H_{(2)} = 1.420$ | ANOVA p = 0.5080<br>TBI WT-Baseline = 0.7155<br>TBI miKO-Baseline > 0.9999<br>TBI miKO-TBI WT > 0.9999 |
| S2 (IL-1α) | 7 mice (Baseline)<br>6 mice (TBI WT)<br>6 mice (TBI miKO) | Normal distribution | One-way ANOVA; Bonferroni *post hoc* | $F_{(2, 16)} = 0.09920$ | ANOVA p = 0.9061<br>TBI WT-Baseline > 0.9999<br>TBI miKO-Baseline > 0.9999<br>TBI miKO-TBI WT > 0.9999 |
| S2 (TECK) | 7 mice (Baseline)<br>6 mice (TBI WT)<br>6 mice (TBI miKO) | Normal distribution | One-way ANOVA; Bonferroni *post hoc* | $F_{(2, 16)} = 0.1090$ | ANOVA p = 0.8974<br>TBI WT-Baseline > 0.9999<br>TBI miKO-Baseline > 0.9999<br>TBI miKO-TBI WT > 0.9999 |
| S2 (IL-17A) | 7 mice (Baseline)<br>6 mice (TBI WT)<br>6 mice (TBI miKO) | Normal distribution | One-way ANOVA; Bonferroni *post hoc* | $F_{(2, 16)} = 0.2218$ | ANOVA p = 0.8035<br>TBI WT-Baseline > 0.9999.<br>TBI miKO-Baseline > 0.9999<br>TBI miKO-TBI WT > 0.9999 |
| S2 (IL13) | 7 mice (Baseline)<br>6 mice (TBI WT)<br>6 mice (TBI miKO) | Non-normal distribution | One-way ANOVA on ranks (Kruskal-Wallis test); Dunn's *post hoc* | $H_{(2)} = 9.129$ | ANOVA p = 0.0051<br>TBI WT-Baseline = 0.0906<br>TBI miKO-Baseline > 0.9999<br>TBI miKO-TBI WT = 0.0104 |
| S2 (SDF-1α) | 7 mice (Baseline)<br>6 mice (TBI WT) | Normal distribution | One-way ANOVA; Bonferroni *post hoc* | $F_{(2, 16)} = 5.197$ | ANOVA p = 0.0182<br>TBI WT-Baseline = 0.7182<br>TBI miKO-Baseline= 0.1610<br>TBI miKO-TBI WT = 0.0173 |

| | | | | | |
|---|---|---|---|---|---|
| | 6 mice (TBI miKO) | | | | |
| S2 (GM-CSF) | 7 mice (Baseline)<br>6 mice (TBI WT)<br>6 mice (TBI miKO) | Normal distribution | One-way ANOVA; Bonferroni *post hoc* | $F_{(2, 16)} = 20.23$ | ANOVA p < 0.0001<br>TBI WT-Baseline = 0.2146<br>TBI miKO-Baseline= 0.0011<br>TBI miKO-TBI WT < 0.0001 |

**References**

1. Wang R, Pu H, Ye Q, Jiang M, Chen J, Zhao J, et al. Transforming Growth Factor Beta-Activated Kinase 1-Dependent Microglial and Macrophage Responses Aggravate Long-Term Outcomes After Ischemic Stroke. Stroke. 2020;51(3):975-85.

2. Aggarwal A, Sharma N, Sandhir R, Rishi V. S-nitrosoglutathione prevents cognitive impairment through epigenetic reprogramming in ovariectomised mice. Biochem Pharmacol. 2019;168:352-65.

3. Ishikawa K, Yoshida S, Nakao S, Sassa Y, Asato R, Kohno R, et al. Bone marrow-derived monocyte lineage cells recruited by MIP-1beta promote physiological revascularization in mouse model of oxygen-induced retinopathy. Lab Invest. 2012;92(1):91-101.
