# Supplementary figures and images for: Microglia-specific deletion of histone deacetylase 3 promotes inflammation resolution, white matter integrity, and functional recovery in a mouse model of traumatic brain injury
Source: J Neuroinflammation. 2022 Aug 6;19:201. doi: 10.1186/s12974-022-02563-2 (PMC9357327; doi:10.1186/s12974-022-02563-2)

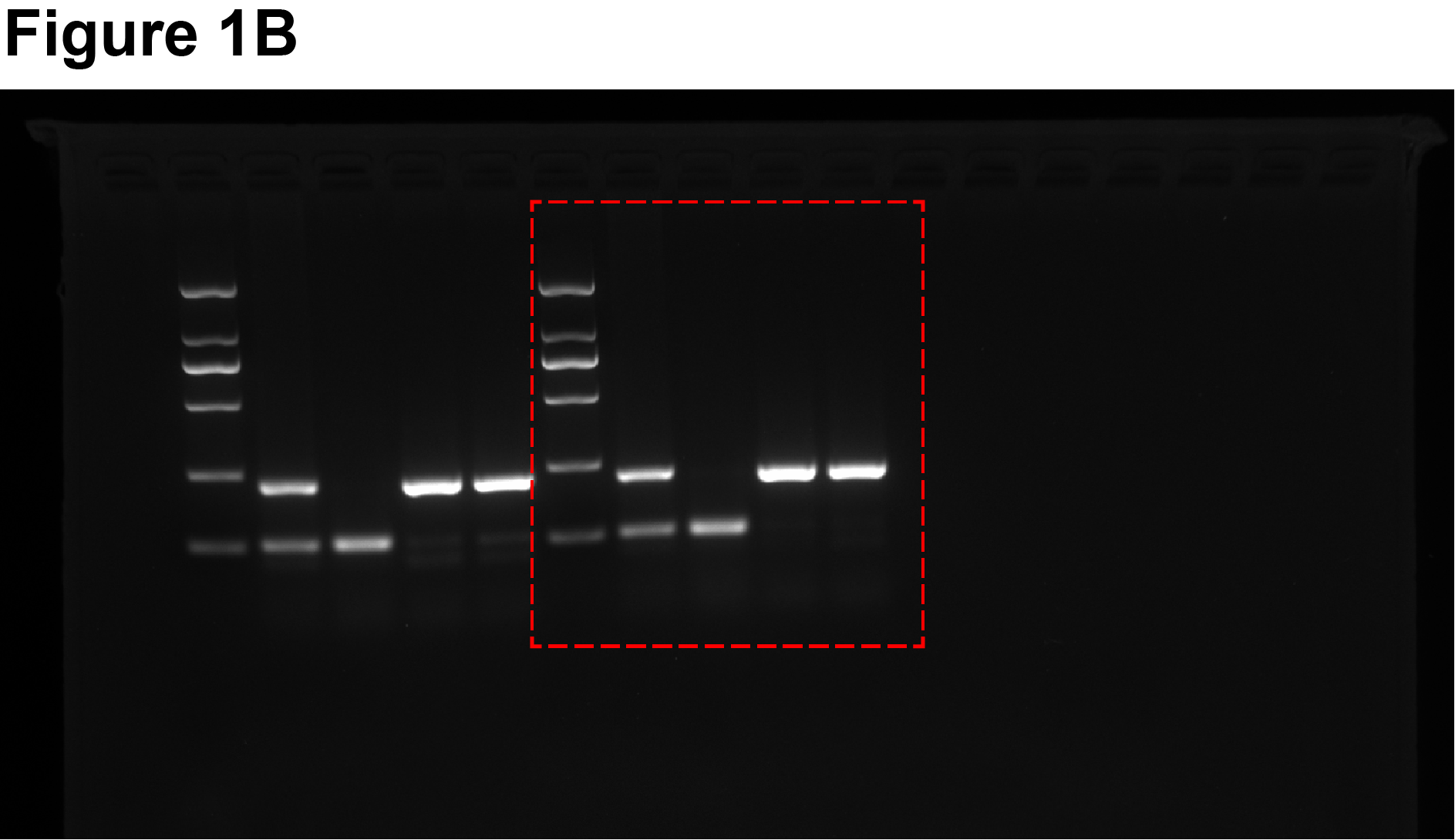

Supplement: Supplementary file 2 — Additional file 2. Raw image of agarose gel in Fig. 1B. [file 12974_2022_2563_MOESM2_ESM.tif]
